# Assessing Wheat Traits by Spectral Reflectance: Do We Really Need to Focus on Predicted Trait-Values or Directly Identify the Elite Genotypes Group?

**DOI:** 10.3389/fpls.2017.00280

**Published:** 2017-03-09

**Authors:** Miguel Garriga, Sebastián Romero-Bravo, Félix Estrada, Alejandro Escobar, Iván A. Matus, Alejandro del Pozo, Cesar A. Astudillo, Gustavo A. Lobos

**Affiliations:** ^1^Facultad de Ciencias Agrarias, Plant Breeding and Phenomic Center, PIEI Adaptación de la Agricultura al Cambio Climático, Universidad de TalcaTalca, Chile; ^2^CRI-Quilamapu, Instituto de Investigaciones AgropecuariasChillán, Chile; ^3^Department of Computer Science, Faculty of Engineering, Universidad de TalcaCuricó, Chile

**Keywords:** phenomic, high-throughput phenotyping, phenotyping, carbon isotope discrimination, reflectance

## Abstract

Phenotyping, via remote and proximal sensing techniques, of the agronomic and physiological traits associated with yield potential and drought adaptation could contribute to improvements in breeding programs. In the present study, 384 genotypes of wheat (*Triticum aestivum* L.) were tested under fully irrigated (FI) and water stress (WS) conditions. The following traits were evaluated and assessed via spectral reflectance: Grain yield (GY), spikes per square meter (SM2), kernels per spike (KPS), thousand-kernel weight (TKW), chlorophyll content (SPAD), stem water soluble carbohydrate concentration and content (WSC and WSCC, respectively), carbon isotope discrimination (Δ^13^C), and leaf area index (LAI). The performances of spectral reflectance indices (SRIs), four regression algorithms (PCR, PLSR, ridge regression RR, and SVR), and three classification methods (PCA-LDA, PLS-DA, and *k*NN) were evaluated for the prediction of each trait. For the classification approaches, two classes were established for each trait: The lower 80% of the trait variability range (Class 1) and the remaining 20% (Class 2 or elite genotypes). Both the SRIs and regression methods performed better when data from FI and WS were combined. The traits that were best estimated by SRIs and regression methods were GY and Δ^13^C. For most traits and conditions, the estimations provided by RR and SVR were the same, or better than, those provided by the SRIs. PLS-DA showed the best performance among the categorical methods and, unlike the SRI and regression models, most traits were relatively well-classified within a specific hydric condition (FI or WS), proving that classification approach is an effective tool to be explored in future studies related to genotype selection.

## Introduction

Wheat is one of the most important cereals in the human diet worldwide. This cereal is consumed in different types of processed foods, providing around 20% of total daily calories (Shewry, 2009). Due to world population growth, it is expected that current wheat production will need to be doubled by the middle of the century (Tilman et al., 2011; FAO, 2015). To accomplish this level of production, wheat yield must increase by 1.60% per year (Dixon et al., 2009), which is far from the 1.26% increase that was reached during the last decade (FAOSTAT, 2013). Additionally, the current effects of climate change on weather patterns, and unexpected events, are threatening maximum thresholds in many areas (Ayeneh et al., 2002; Azimi et al., 2010; Rebetzke et al., 2012; Hernández-Barrera et al., 2016).

This challenging scenario should encourage wheat breeders to accelerate the development and release of new high-yield cultivars that are adapted to more complex environmental conditions (Velu and Prakash, 2013). One strategy for improving and expediting the selection of these elite genotypes is the acquisition of high-dimensional phenotypic data (high-throughput phenotyping) (Bowman et al., 2015; Camargo and Lobos, 2016; Crain et al., 2016).

Remote sensing techniques, such as spectrometry, are increasingly used for plant phenotyping (Cabrera-Bosquet et al., 2012; Araus and Cairns, 2014). Spectral reflectance or the spectrum of energy reflected by the plant is closely associated with absorption at certain wavelengths that are linked to specific characteristics or plant conditions (Lobos and Hancock, 2015). Spectrometers can acquire detailed information regarding the electromagnetic spectrum in a short time, making this technology ideal for assessing hundreds or thousands of genotypes within a few hours (Cabrera-Bosquet et al., 2012). This would enable researchers and breeders to estimate multiple morpho-physiological and physico-chemical traits, which would otherwise be impossible to evaluate due to the time and cost involved (Lobos and Hancock, 2015). This would be reflected in reduced breeding program costs and, by allowing for the early selection of genetic material of interest, increase the chances of releasing improved cultivars in less time (Lobos and Hancock, 2015; Camargo and Lobos, 2016).

For the estimation of wheat traits, such as grain yield, biomass, leaf area index, plant height, and isotopic carbon discrimination, the majority of previous studies have resorted primarily to the use of “Spectral Reflectance Indices” (SRIs) (Aparicio et al., 2002; Babar et al., 2006a,b; Marti et al., 2007; Prasad et al., 2007; Gutierrez et al., 2010; Lobos et al., 2014; Hernández et al., 2015), whereas there has been less attention paid to the development of multivariate regression models, using part or all of the spectral reflectance (Pimstein et al., 2011; Dreccer et al., 2014; Li F. et al., 2014; Li X. et al., 2014; Hernández et al., 2015; Siegmann and Jarmer, 2015; Yao et al., 2015).

Current research into plant phenotyping and phenomics for plant breeding has focused on using the spectral signature to estimate predicted trait-values rather than exploring other tools that could directly identify elite genotypes for the desired trait. The use of reflectance data and categorical methods for breeding purposes has been scarcely addressed by the scientific community. Nonetheless, some successful experiments have been carried out: To classify lines for waxy alleles in durum wheat (Delwiche et al., 2006; Lavine et al., 2014) and bread wheat (Delwiche et al., 2011); to identify wheat lines possessing wheat-rye translocations (Delwiche et al., 1999); to classify barley varieties (Porker et al., 2017); and to select haploid kernels from hybrid kernels in maize (Jones et al., 2012).

The aim of the present study was, based on plant reflectance data, to assess the feasibility of using a categorical approximation to select featured genotypes, by comparing the performance of a large set of SRIs, multivariate regression models (PCR, PLSR, ridge regression, and SVR), and categorical models (PCA-LDA, PLS-DA, and *k*NN) in the prediction of grain yield, agronomic yield components, and physiological traits.

## Materials and methods

### Plant material and experimental conditions

A set of 384 cultivars and advanced lines of spring bread wheat (*Triticum aestivum* L.) with good agronomic characteristics and disease tolerance were evaluated (list at del Pozo et al., 2016). These genotypes were sourced from three wheat-breeding programs (INIA-Chile, INIA-Uruguay, and CIMMYT-Mexico) and are currently being used to breed for adaptation to drought stress and to develop suitable genotypes for wheat production in the drylands of Chile and other countries.

Experiments were conducted in 2012 in two contrasting Mediterranean environments of Chile: Cauquenes (35°58′S, 72°17′W), with typical rain-fed conditions such that the plants were grown under water stress (WS); and Santa Rosa (36°32′S, 71°55′W), under fully irrigated (FI) conditions. The precipitation in these locations during the experiments was 183 and 700 mm, respectively (Table 1). Under FI conditions, four furrow irrigations of approximately 50 mm each were applied at the end of tillering (Zadocks Stage 21; Z21), the flag leaf stage (Z37), heading (Z50), and middle grain filling (Z70) (Zadoks et al., 1974). There was a difference of 28 days (77–105 days) between the earlier and later genotypes in reaching the heading stage; 89% of the genotypes 81–94 days.

**Table 1:** **Monthly maximum, minimum, and mean temperature, and monthly rainfall at the two experimental sites in central Chile during the trial (May 2012–January 2013)**.

**Cauquenes**		**May**	**Jun**.	**Jul**.	**Aug**.	**Sept**.	**Oct**.	**Nov**.	**Dec**.	**Jan**.
Temp. (°C)	Max.	18.1	14.1	15.8	14.7	19.6	19.9	25.7	24.6	30.8
	Min.	5.3	5.8	4.5	5.5	6.6	5.9	8.5	9.3	12.5
	Mean	11.7	10.0	11.6	11.3	15.0	12.8	16.8	17.0	21.2
Rainfall (mm)		10.0	9.0	14.0	22.8	3.0	24.5	69.6	29.7	0.0
**Santa Rosa**		**May**	**Jun**.	**Jul**.	**Aug**.	**Sept**.	**Oct**.	**Nov**.	**Dec**.	**Jan**.
Temp. (°C)	Max.	16.2	13.1	12.6	13.2	18.2	18.9	24.2	23.1	30.0
	Min.	4.4	5.1	0.8	3.5	4.1	5.3	7.5	8.1	11.1
	Mean	9.8	8.6	6.1	7.6	10.6	11.8	15.3	15.6	19.9
Rainfall (mm)		90.0	186.9	52.5	139.3	12.7	48.0	59.2	109.6	1.2

The experiment was conducted in an incomplete block design (α-lattice), with two replicates per genotype (*n* = 384 × 2). Plots had five rows, each 2 m in length, the distance between the rows was 0.2 m, and the distance between plots was 0.4 m. Similar agronomic practices were performed at the two locations. Sowing (20 g m^−2^) dates were 23 May at Cauquenes and 7 August at Santa Rosa. Before sowing, the plots were fertilized with 260 kg ha^−1^ of ammonium phosphate (46% P_2_O_5_ and 18% N), 90 kg ha^−1^ of potassium chloride (60% K_2_O), 200 kg ha^−1^ of sul-po-mag (22% K_2_O, 18% MgO and 22% S), 10 kg ha^−1^ of boronatrocalcite (11% B), and 3 kg ha^−1^ of zinc sulfate (35% Zn). During tillering, an extra 153 kg ha^−1^ of N was applied. Flufenacet + Flurtamone + Diflufenican (96 g a.i.) was applied for pre-emergence weed control and a further application of MCPA (525 g a.i.) + Metsulfuron-metil (5 g a.i.) was used for post-emergence weed control.

### Trait measurements

#### Grain yield and agronomic yield components

The number of spikes per m^2^ (SM2) was determined for a 1 m length of an inside row. The number of kernels per spike (KPS) and thousand-kernel weight (TKW) were determined in 25 spikes selected at random from the central row. Grain yield (GY) was assessed by harvesting the whole plot.

#### Leaf chlorophyll and water-soluble carbohydrate

Chlorophyll (Chl) content was determined using the SPAD index for five flag leaves per plot, at anthesis (*an*) and at grain filling (*gf*), with a portable leaf chlorophyll-meter (SPAD 502, Minolta Spectrum Technologies Inc., Plainfield, IL, USA). Water-soluble carbohydrate was determined using the anthrone reactive method (Yemm and Willis, 1954), for five main stems (excluding leaf laminas and sheaths) per plot, at *an* and at maturity (*m*), and expressed as concentration (WSC, mg g^−1^ DW) and content (WSCC, mg stem^−1^).

#### Carbon isotope discrimination

For kernels sampled randomly at *m*, the stable carbon (^13^C/^12^C) isotope ratio was measured using an elemental analyzer (ANCA-SL, PDZ Europa, UK) coupled with an isotope ratio mass spectrometer, at the Laboratory of Applied Physical Chemistry at Ghent University (Belgium). The carbon isotope discrimination (Δ^13^C) was calculated as follows: Δ^13^C (‰) = (δ^13^C_*a*_–δ^13^C_*p*_)/[1+ (δ^13^C_*p*_)/1000], where *a* and *p* refer to air and plant, respectively (Farquhar et al., 1989). δ^13^C_a_ was taken as 8.0‰.

#### Leaf area index

The leaf area index (LAI) at *an* (under FI conditions only) was determined by measuring the incident light falling on the crop and the amount of light in each plot at ground level, using a BF5 Sunshine Sensor and SunScan Canopy Analyser (Delta-T, Cambridge, UK). The radiation transmitted and dispersed by the canopy was recorded, and the LAI then calculated.

### Spectral reflectance measurements

Canopy spectral reflectance (350–2,500 nm) was measured using a portable spectroradiometer (FieldSpec 3 JR, ASD, Boulder, CO, USA) at two developmental stages: Anthesis (*AN*; denoted with capital letters to avoid confusion with trait measurements stages) and grain filling (*GF*). The optical fiber (2.3 mm diameter with 25° full conical angles) was placed 80 cm above the canopy, at a 45° angle. From 11:00 to 17:00 h on clear sunny days, measurements were taken by moving (sweeping) the sensor over the plot, covering the three central rows. The equipment was set up to take three scans per plot and the average for each wavelength was considered in further analyses. To limit variations in reflectance induced by changes in the angle of the sun, radiometric calibration was performed every 15 min, using a white barium sulfate panel as the reference (Spectralon, ASD, Boulder, CO, USA). Prior to modeling, exploratory analysis and spectral noise deletion were performed using the software Spectral Knowledge (SK-UTALCA) (Lobos and Poblete-Echeverría, 2017).

### Modeling analysis

Spectral reflectance assessed at *AN* and *GF* stages was used to estimate the traits that were evaluated at anthesis (*an*) (Chl content, WSC, WSCC, and LAI), grain filling (*gf*) (Chl content), and maturity (*m*) (SM2, KPS, TKW, GY, WSC, WSCC, and Δ^13^C). The following analyses were considered.

#### Spectral reflectance indices

Spectral reflectance was used to assess the predictive performance of a set of 255 SRIs loaded on SK-UTALCA (Lobos and Poblete-Echeverría, 2017). Using the linear regression analysis option, the relationships between the each of the measured traits and each of the SRIs at *AN* and *GF* were examined using the coefficient of determination (*r*^2^) and the root mean square error (RMSE). WS and FI conditions were analyzed independently, but also as one environment (WS+FI).

#### Multivariate regression methods

Four different regression methods were considered: Principal Components Regression (PCR), Partial Least Square Regression (PLSR), Ridge Regression (RR), and Support Vector Machine Regression (SVR). Prior to modeling with R 3.1.2 software (R Development Core Team, 2011), any samples with missing values were excluded, outliers were identified by Local Outlier Detection (LOF), and the data were normalized.

PCR is a combination of Principal Component Analysis (PCA) and Multiple Linear Regression (MLR), which first reduces the dimensionality of the spectral data, concentrating the information into so-called principal components. The transformed data is then used to train a MLR model that fits a linear equation (Jolliffe, 2002).

PLSR reduces the dimensionality of the data by constructing so-called latent factors. Unlike PCA, PLSR produces a set of factors that take into consideration the values of the independent and dependent variables simultaneously. In this sense, PLSR finds vectors that not only represent the variance of the data but that are also related to the response (Wold et al., 2001; Hastie et al., 2005).

RR works in a similar way to least square fitting, but adds a term that penalizes the values of the coefficients. The role of the penalization term is to “shrink” the estimates of the coefficients toward zero (Hastie et al., 2005). This penalization can be controlled using a tuning parameter λ, which has to be estimated independently. The optimization of λ was performed using a grid of 100 possible values of λ in a range of [10^−2^, 10^10^] with 10-fold cross-validation. The best λ identified was used to build the model.

SVR is a method derived from the Support Vector Machine (SVM). The SVM transforms data into a new high-dimensional space using a kernel function. In this newly created space, a predictive model is built using a subset of representative instances called support vectors. SVR estimates a linear dependency by fitting an optimal approximating hyperplane to the training samples in the multidimensional feature space. In the present study, several kernels (linear, polynomial, radial basis function, and sigmoidal) were automatically tested and selected based on a minimization criterion. The parameters *C* (regularization parameter) and ε (loss function parameter) were fixed to 1 and 0.1, respectively.

All models were validated by 10-fold cross-validation (10xCV) and their performances evaluated by the coefficient of determination (*R*^2^), the root mean square error (RMSE), and the Index of Agreement (IA) in calibration and validation. The IA (Willmott, 1981) is a standardized measure for estimating the prediction error of the model and ranges from 0 (faulty model) to 1 (perfect fit).

#### Multivariate classification methods

Spectral reflectance data were also modeled by three different supervised classification methods: Principal Components—Linear Discriminant Analysis (PCA-LDA), Partial Least Square Discriminant Analysis (PLS-DA), and the *k*-Nearest Neighbor (*k*NN) algorithm. Two different categories were established by taking into account the total variation of each trait, as measured at each of the developmental stages and in each of the environments (FI, WS, or WS+FI). The first category, labeled as “Class 1,” corresponds to instances with values in the lower 80% of the trait range. The remaining 20% of instances were considered as belonging to the elite group and were labeled as “Class 2” (Supplementary Table 1). The goal of this dichotomization was to develop predictive models that were able to identify those elite genotypes that had the highest trait performance (the upper 20% of the trait range). Model calculations were done using the Classification Tool Box (Version 4.2) developed by Milano Chemometrics and the QSAR Research Group (Ballabio and Consonni, 2013) and implemented in Matlab 8.2.0 (The Math Works Inc., MA, USA).

PCA-LDA is a classification technique based on the linear discriminant functions. PCA is used to reduce the dimensionality of the spectral matrix and LDA acts as the classifier. Classes are separated by maximizing the variance between the groups, and minimizing the variance within the groups, to determine the best fit of parameters for the classification (Lehmann et al., 2015). Before calculating the PCA-LDA models, the input data were mean-centered and the optimal number of principal components was searched in the interval 1–20, with 10xCV, on the basis of minimizing the error rate of validation. The discrimination of classes was established as linear.

PLS-DA is a pattern recognition method that combines the properties of PLSR, discriminating between the categories using the Discriminant Analysis technique (Ballabio and Consonni, 2013). PLS-DA works by finding the latent variables that describe the variance in both the independent X variables (spectra) and the dependent Y variables (classes) and are able to separate the data into two or more classes (Barker and Rayens, 2003). In PLS-DA, a model is developed for each class and the probability that a sample belongs to a specific class is calculated based on the estimated class values (Ballabio and Consonni, 2013). In the present study, the PLS-DA models were calculated using mean-centered data and the optimal number of latent variables was searched in the range [1, 20], with 10xCV.

Finally, the k-nearest neighbors (*k*NN) method is based on the determination of the distances between an instance whose identity is assumed to be unknown and each instance belonging to the training set. Once the distances are computed, the elements are ranked according to their proximity to the query instance, selecting the *k* elements that are closest to this. Finally, the category of the query item is estimated using a majority voting scheme among the labels of the *k* selected items (Cunningham and Delany, 2007). In general, the distance function can be any mathematical function that expresses dissimilarity but, for simplicity, a common choice is the Euclidian distance. In this study, the data was mean-centered and the best value for the parameter *k* was obtained from values in [1,10], with 10xCV.

The predictive powers of the categorical PCA-LDA, PLS-DA, and *k*NN models were evaluated by calculations of accuracy, error rate, and prediction rates (determined by the sensibility of each class) for both classes in calibration and validation.

## Results

The range of values, and their means, for each of the traits evaluated in the 384 wheat genotypes grown under FI and WS conditions, are presented in Table 2.

**Table 2:** **Traits evaluated for 384 genotypes of wheat grown under fully irrigated (FI) and water stress (WS) conditions, in 2012**.

**Trait^y^**	**FI**		**WS**	
	**Range**	**Mean ± SD**	**Range**	**Mean ± SD**
SM2*m*^z^	320.0–1125.0	627.7 ± 133.7	75.0–625.0	321.1 ± 69.3
KPS*m*	15.0–68.9	38.6 ± 6.5	9.4–55.6	32.3 ± 6.3
TKW*m* (g)	30.8–88.6	49.3 ± 6.6	29.4–89.4	44.4 ± 6.8
GY*m* (ton ha^−1^)	5.1–12.9	9.7 ± 1.2	0.9–6.9	3.1 ± 0.9
Chl*an* (SPAD index)	35.9–58.2	49.3 ± 3.3	31.3–52.0	41.4 ± 3.5
Chl*gf* (SPAD index)	30.1–56.0	47.8 ± 3.7	0.8–48.8	34.0 ± 10.3
WSC*an* (mg g^−1^ DW)	16.1–610.9	141.2 ± 49.2	20.6–708.4	226.8 ± 51.7
WSC*m* (mg g^−1^ DW)	5.6–686.3	43.4 ± 33.3	5.0–218.3	48.0 ± 23.5
WSCC*an* (mg stem^−1^)	19.3–926.0	172.1 ± 86.3	32.7–1271.6	410.8 ± 140.9
WSCC*m* (mg stem^−1^)	6.1–926.6	47.4 ± 45.1	4.9–262.9	52.1 ± 34.4
Δ^13^C*m* (‰)	17.1–20.2	18.8 ± 0.5	12.3–16.5	14.9 ± 0.5
LAI*an*	2.4–8.4	5.2 ± 1.0	–	−

y *SM2: spikes m^−2^; KPS, kernels spike^-1^; TKW, thousand kernels weight; GY, grain yield; Chl, SPAD index; water soluble carbohydrates concentration (WSC) and content (WSCC); Δ^13^C, isotopic discrimination of ^13^C; LAI, leaf area index*.

z *Trait measurement at anthesis (an), grain filling (gf), or maturity (m)*.

### Spectral reflectance indices

Coefficients of determination greater than 0.8 were only reached when the hydric conditions were combined (WS+FI) for GY*m* (*AN*: 0.82 and *GF*: 0.92) and Δ^13^C*m* (*AN*: 0.82 and *GF*: 0.92) (Table 3; Supplementary Table 2). Among the 255 SRIs tested, NWI-3 [(R_970_–R_920_)/(R_970_+R_920_) worked at *AN* and WI2 (R_970_/R_900_) worked at *GF*. When the hydric conditions were kept separate, predictions with *r*^2^ values greater than 0.25 were achieved only for WS conditions and spectral measurements performed at *GF* (NWI-3; GY*m*: 0.51 and Δ^13^C*m*: 0.26).

**Table 3:** **Best spectral reflectance indices (SRI), regression and classification models calculated by trait, reflectance assessment and hydric condition**.

**Trait^u^**	**Reflectance assessment^w^**	**Hydric condition^x^**	**Spectral reflectance indices**		**Regression models**				**Classification models**		
					**PCR**	**PLSR**	**RR**	**SVR**	**PCA-LDA**	**PLS-DA**	***k*****NN**
			**SRI^y^**	***r^2^***	***R^2^***	***R^2^***	***R^2^***	***R^2^***	**Pred. Rate (Class 2)**	**Pred. rate (Class 2)**	**Pred. rate (Class 2)**
SM2*m*^v^	AN	WS	SR (550;670)	0.15^*z*^	0.11	0.12	0.12	0.11	0.23	0.55	0.17
		FI	NRI (1510;660)	0.24	0.23	0.25	0.34	0.26	0.41	0.71	0.30
		WS+FI	MTCI (800;750;670)	0.63	0.39	0.59	0.73	0.73	0.57	0.87	0.50
	GF	WS	CI (415;695)	0.11	0.03	0.10	0.20	0.15	0.18	0.64	0.29
		FI	NDSI (403;830)	0.20	0.18	0.18	0.24	0.20	0.23	0.68	0.21
		WS+FI	SAVI (807;736)	0.66	0.59	0.68	0.75	0.74	0.51	0.90	0.46
KPS*m*	AN	WS	NDSI (543;548)	0.03	0.01	0.01	0.06	0.05	0.04	0.43	0.09
		FI	RBI (695;445)	0.10	0.03	0.07	0.13	0.16	0.06	0.47	0.12
		WS+FI	NDSI (543;548)	0.19	0.15	0.16	0.20	0.22	0.07	0.71	0.27
	GF	WS	NDSI (933;948)	0.13	0.05	0.07	0.13	0.14	0.07	0.59	0.22
		FI	SR (690;655)	0.10	0.03	0.07	0.07	0.05	0.03	0.59	0.26
		WS+FI	SR (960;950)	0.24	0.21	0.21	0.24	0.26	0.07	0.73	0.28
TKW*m*	AN	WS	SR (690;655)	0.13	0.06	0.08	0.08	0.07	0.05	0.69	0.16
		FI	NRI (1510;660)	0.29	0.30	0.30	0.30	0.30	0.21	0.82	0.35
		WS+FI	NRI (1510;660)	0.27	0.21	0.21	0.26	0.28	0.25	0.73	0.39
	GF	WS	WI (950;900)	0.10	0.02	0.06	0.21	0.27	0.24	0.69	0.30
		FI	NRI (1510;660)	0.15	0.16	0.17	0.20	0.19	0.26	0.74	0.30
		WS+FI	WI (950;900)	0.16	0.17	0.18	0.33	0.38	0.24	0.80	0.33
GY*m*	AN	WS	PRI * CI (570;530;760;700)	0.09	0.07	0.11	0.14	0.19	0.18	0.68	0.22
		FI	SR (440;685)	0.12	0.02	0.10	0.16	0.23	0.10	0.61	0.24
		WS+FI	NDWI (970;920)	0.82	0.63	0.68	0.89	0.90	0.54	0.93	0.44
	GF	WS	NDWI (970;920)	0.51	0.33	0.36	0.48	0.56	0.49	0.78	0.32
		FI	WI (900;970)	0.14	0.09	0.11	0.14	0.15	0.12	0.69	0.18
		WS+FI	WI (970;900)	0.92	0.83	0.85	0.92	0.93	0.53	0.98	0.51
Chl*an*	AN	WS	PRI (550;531)	0.09	0.00	0.09	0.09	0.10	0.06	0.65	0.25
		FI	MCARI (700;670;550)	0.18	0.07	0.17	0.21	0.20	0.03	0.63	0.16
		WS+FI	TCARI (700;600;550;850;670)	0.59	0.48	0.53	0.58	0.59	0.41	0.96	0.45
	GF	WS	AI (740;887;691;698)	0.14	0.01	0.12	0.26	0.25	0.20	0.63	0.17
		FI	PRI (512;531)	0.11	0.02	0.06	0.07	0.07	0.06	0.75	0.22
		WS+FI	MTCI (800;750;670)	0.60	0.51	0.55	0.61	0.66	0.41	0.97	0.41
Chl*gf*	AN	WS	NDSI (410;550)	0.04	0.00	0.04	0.07	0.05	0.10	0.71	0.23
		FI	MCARI (700;670;550)	0.21	0.07	0.14	0.14	0.11	0.14	0.80	0.27
		WS+FI	NDWI (970;920)	0.42	0.34	0.38	0.42	0.44	0.57	0.95	0.50
	GF	WS	SR (690;655)	0.05	0.04	0.05	0.11	0.11	0.18	0.66	0.18
		FI	PRI (512;531)	0.14	0.04	0.10	0.13	0.12	0.16	0.71	0.17
		WS+FI	NDWI (970;920)	0.44	0.42	0.43	0.44	0.51	0.52	0.96	0.48
WSC*an*	AN	WS	NDSI (1060;1118)	0.05	0.02	0.05	0.09	0.10	0.03	0.53	0.26
		FI	SAVI (807;736)	0.12	0.10	0.13	0.19	0.11	0.20	0.63	0.16
		WS+FI	MTCI (800;750;670)	0.41	0.23	0.30	0.49	0.48	0.29	0.85	0.36
	GF	WS	RE (670;780)	0.04	0.02	0.03	0.07	0.06	0.03	0.59	0.19
		FI	SAVI 2 (800;670)	0.09	0.08	0.08	0.09	0.10	0.10	0.63	0.29
		WS+FI	NDSI (933;948)	0.44	0.37	0.41	0.46	0.49	0.22	0.87	0.31
WSC*m*	AN	WS	NDTI (1650;2215)	0.06	0.03	0.04	0.06	0.03	0.11	0.55	0.22
		FI	WDVI (830;660)	0.04	0.00	0.01	0.01	0.01	0.01	0.51	0.27
		WS+FI	NDTI (1650;2215)	0.03	0.01	0.02	0.03	0.03	0.04	0.52	0.26
	GF	WS	NDSI (940;1122)	0.07	0.03	0.03	0.07	0.06	0.15	0.59	0.17
		FI	NDSI (1060;1118)	0.03	0.00	0.02	0.02	0.03	0.06	0.50	0.22
		WS+FI	SR (960;950)	0.03	0.00	0.01	0.04	0.05	0.02	0.54	0.17
WSCC*an*	AN	WS	NRI (1510;660)	0.05	0.03	0.04	0.07	0.06	0.05	0.56	0.03
		FI	NRI (1510;660)	0.20	0.21	0.23	0.30	0.22	0.24	0.78	0.22
		WS+FI	MTCI (800;750;670)	0.47	0.29	0.43	0.55	0.53	0.28	0.92	0.42
	GF	WS	NDSI (442;438)	0.04	0.03	0.03	0.10	0.07	0.03	0.66	0.23
		FI	SAVI 2 (800;670)	0.15	0.15	0.15	0.18	0.16	0.18	0.70	0.31
		WS+FI	WI (970;900)	0.50	0.40	0.46	0.56	0.56	0.31	0.90	0.31
WSCC*m*	AN	WS	NDTI (1650;2215)	0.06	0.03	0.04	0.06	0.05	0.09	0.66	0.20
		FI	WDVI (830;660)	0.04	0.00	0.00	0.02	0.02	0.01	0.54	0.25
		WS+FI	NDTI (1650;2215)	0.02	0.02	0.02	0.03	0.03	0.05	0.61	0.28
	GF	WS	NDWI (870;1260)	0.12	0.08	0.09	0.13	0.08	0.13	0.61	0.14
		FI	BI (460;660)	0.03	0.00	0.01	0.02	0.02	0.05	0.56	0.23
		WS+FI	NDSI (503;483)	0.03	0.01	0.01	0.06	0.06	0.07	0.58	0.17
Δ^13^C*m*	AN	WS	TCARI (700;670;550)	0.11	0.05	0.10	0.10	0.15	0.25	0.58	0.20
		FI	SIPI (800;440;680)	0.05	0.01	0.02	0.11	0.12	0.10	0.58	0.13
		WS+FI	NDWI (970;920)	0.82	0.63	0.68	0.91	0.92	0.40	0.89	0.41
	GF	WS	NDWI (970;920)	0.26	0.12	0.16	0.23	0.34	0.42	0.66	0.27
		FI	NDWI (970;850)	0.06	0.02	0.03	0.10	0.10	0.04	0.58	0.22
		WS+FI	WI (970;900)	0.92	0.81	0.85	0.93	0.94	0.43	0.95	0.40
LAI*an*	AN	FI	Datt (850;710;680)	0.44	0.36	0.41	0.37	0.45	0.63	0.82	0.54
	GF	FI	SAVI 2 (800;670)	0.32	0.26	0.33	0.36	0.37	0.46	0.78	0.34

u *SM2, spikes m^−2^; KPS, kernels spike^−1^; TKW, thousand kernels weight; GY, grain yield; Chl, SPAD index; water soluble carbohydrates concentration (WSC) and content (WSCC); Δ^13^C, isotopic discrimination of ^13^C; LAI, leaf area index*.

v *Trait measurement at anthesis (an), grain filling (gf), or maturity (m)*.

w *Spectral reflectance measurement at anthesis (AN) and grain filling (GF)*.

x *Hydric conditions were water stress (WS), full irrigated (FI), and the combination (WS+FI)*.

y *Spectral Reflectance Index: List at Lobos and Poblete-Echeverría (2017)*.

z *In bold values (R^2^ or prediction rate) between 0.40 and 0.79 (red) and ≥0.80 (blue)*.

Coefficients of determination between 0.40 and 0.79 were found with combined hydric conditions for the following traits: SM2*m* [*AN* MTCI ((R_800_–R_750_)/(R_750_–R_670_)): 0.63; *GF* SAVI2 (1.5*(R_807_–R_736_)/(R_807_+R_736_+0.5)): 0.66]; Chl*an* [*AN* TCARI2 (3*((R_700_–R_600_)−((0.2*(R_700_–R_550_))*(R_700_/(R_850_+R_670_)))): 0.59; *GF* MTCI: 0.60]; Chl*gf* [*AN* NWI-3: 0.42; *GF* NWI-3: 0.44]; WSC*an* [*AN* MTCI: 0.41; *GF* NDSI4 ((R_933_–R_948_)/(R_933_+R_948_)): 0.44]; and WSCC*an* (*AN* MTCI: 0.47; *GF* WI2: 0.50). When the hydric conditions were kept separate, *r*^2^ values between 0.4 and 0.79 were found only for LAI*an* under FI conditions [*AN* DATT ((R_850_–R_710_)/(R_850_–R_680_): 0.44] (Table 3).

### Multivariate regression methods

As observed for the SRIs, the four multivariate regression models (PCR, PLSR, RR, and SVR) showed the highest predictive power for most traits when data from both hydric conditions were combined (WS+FI), with the exception of TKW*m* at *AN* under FI conditions (Table 3, Supplementary Figure 1, and Supplementary Table 2). The $R_{cv}^{2}$ values were similar between RR and SVR, and greater than those for the other two multivariate models. Using RR or SVR, $R_{cv}^{2}$ values greater than 0.8 were found for GY*m* (*AN*: 0.90 and *GF*: 0.93) and Δ^13^C*m* (*AN*: 0.92 and *GF*: 0.94). In addition, $R_{cv}^{2}$ values between 0.40 and 0.79 were found for the following traits when using SVR with combined hydric conditions: SM2*m* (*AN*: 0.73 and *GF*: 0.74), Chl*an* (*AN*: 0.59 and *GF*: 0.66), Chl*gf* (*AN*: 0.44 and *GF*: 0.51), WSC*an* (*AN*: 0.48 and *GF*: 0.49), and WSCC*an* (*AN*: 0.53 and *GF*: 0.56). When the hydric conditions were kept separate, *r*^2^ values in this same range were achieved only for GY*m* (*GF*: 0.56) under WS conditions, and for LAI (*AN*: 0.45) under FI conditions (Table 3).

### Multivariate classification methods

**Figure 1:**
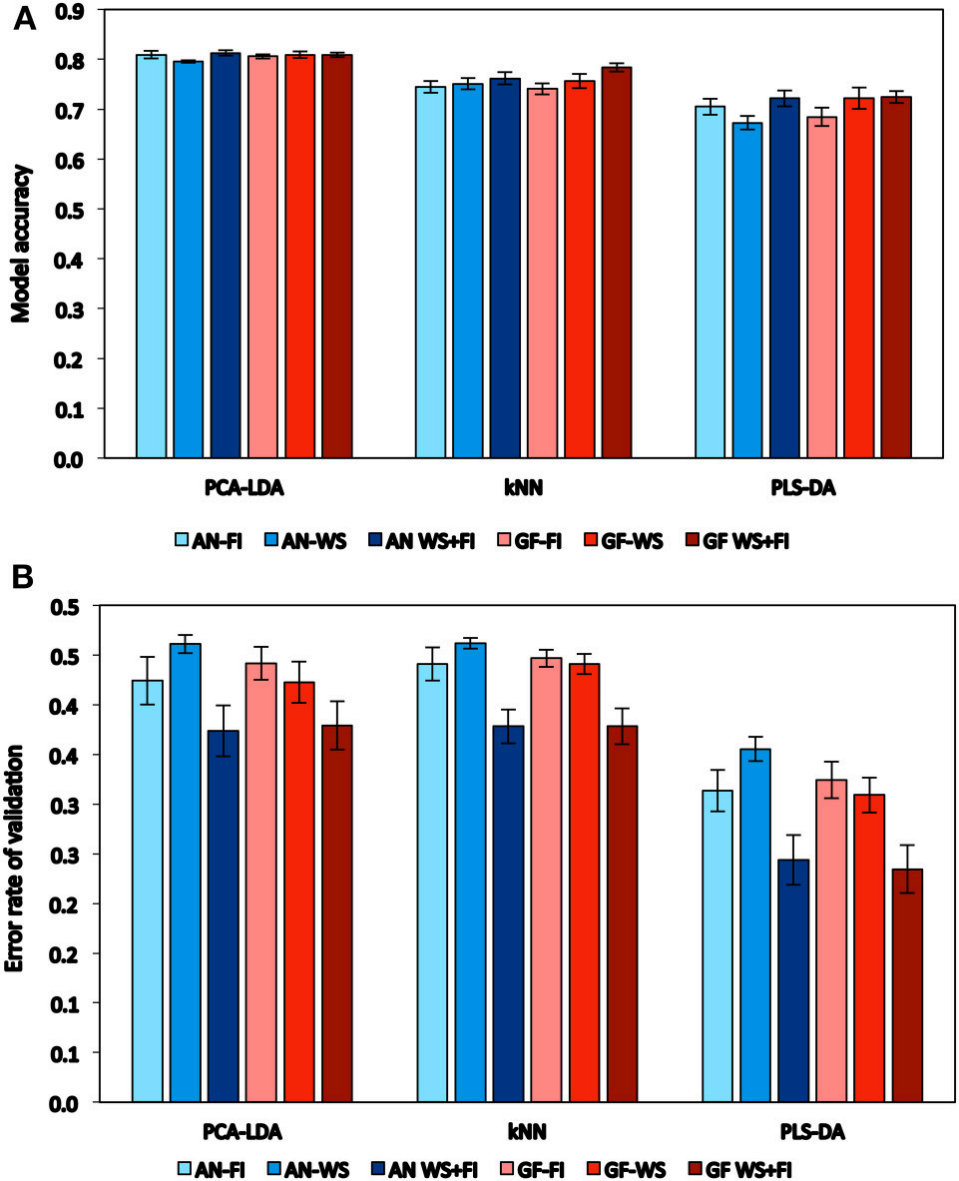
**Performance of classification models on the basis of the average of model accuracy (A)** and error rate of cross-validation **(B)** calculated to all traits and estimated by spectral reflectance at anthesis (*AN*) and grain filling (*GF*). Wheat genotypes growing under two hydric conditions (FI: fully irrigated and WS: water stress); combination of both conditions (WS+FI) for modeling purposes. Vertical bars represent the standard error.

The general performances of the categorical models were very similar in terms of model accuracy with 10xCV (Figure 1A); PCA-LDA, *k*NN, and PLS-DA showed average accuracies of 0.81, 0.76, and 0.71, respectively. PLS-DA, however, showed the lowest error rate of validation (Figure 1B); the average errors were approximately 0.42, 0.42, and 0.30 for PCA-LDA, *k*NN, and PLS-DA, respectively. Moreover, the error rate of validation showed differences within each model, being similar for the two hydric conditions when these were kept separate, but higher when the WS and FI conditions were combined.

**Figure 2:**
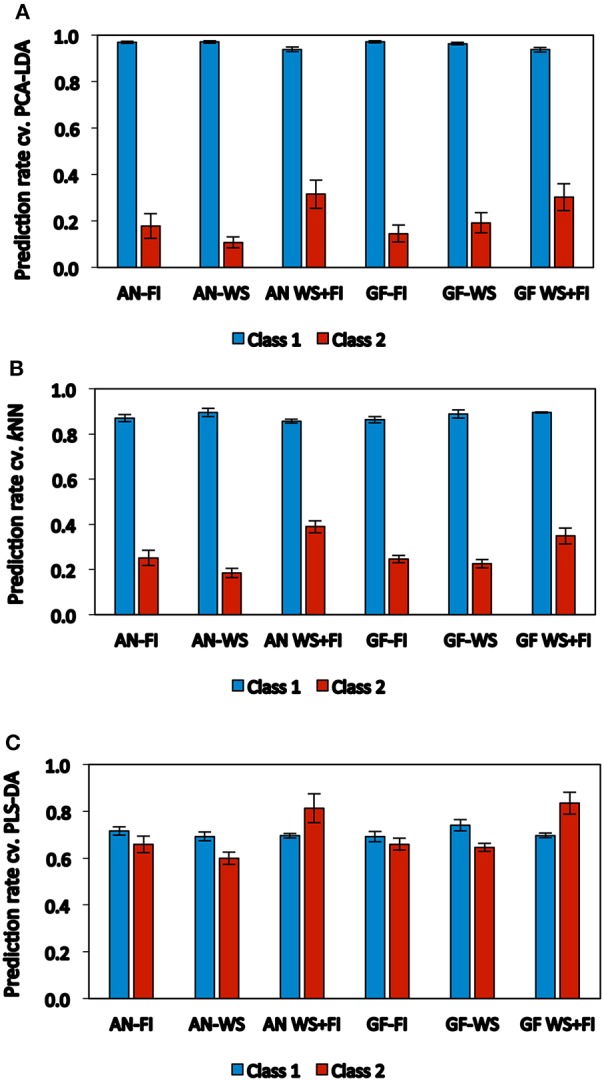
**Performance of each classification method** (**A**: PCA-LDA, **B**: *k*NN, and **C**: PLS-DA) on the basis of the average of prediction rate of cross-validation calculated to all traits and classes, and estimated by spectral reflectance at anthesis (*AN*) and grain filling (*GF*). Wheat genotypes growing under two hydric conditions (FI: fully irrigated and WS: water stress); combination of both conditions (WS+FI) for modeling purposes. Vertical bars represent the standard error.

The general performance of the three categorical models was evaluated based on the prediction rate of cross-validation for both classes (Figure 2), which proved to be different between models. PCA-LDA and *k*NN showed greater prediction levels for samples included in Class 1 (0.96 and 0.88, respectively), but very low prediction levels for samples from Class 2 (0.21 and 0.27, respectively) (Figures 2A, B). Meanwhile, PLS-DA showed lower prediction levels for Class 1, however, the prediction rates were similar for both Class 1 (~0.70) and Class 2 (~0.71) (Figure 2C).

Considering the prediction rates for both classes, the best genotype discriminations were obtained for most traits by PLS-DA when both hydric conditions were combined, with the exception of WSC*m* and WSCC*m*, at WS (*AN* and *GF*). Prediction rates from cross-validation by PLS-DA for Class 1 under WS+FI conditions ranged from 0.64 (KPS*m* at *AN* and *GF*, and WSCC*m* at *AN*) to 0.77 (SM2*m* at *AN* and *GF*), while prediction rates for Class 2 were between 0.52 (WSC*m* at *AN*) and 0.98 (GY at *GF*). However, the prediction rates for several traits were greater when the hydric conditions were kept separate, when compared to those rates achieved with combined WS+FI conditions. This was observed mainly for Class 1 (e.g., KPS*m AN*-FI, GY*m GF*-WS, and Δ^13^C*m GF*-WS) but also for Class 2 (e.g., TKW*m AN*-FI, WSC*m GF*-WS, and WSCC*m AN*-WS).

Importantly, unlike the SRI and multivariate regression methods, the PLS-DA model generated high prediction levels for the individual hydric conditions, in both classes, for most of the traits evaluated. Under FI conditions, the prediction rates for Class 1 ranged from 0.56 (Chl*an* at *GF*) to 0.85 (LAI*an* at *AN*), while the prediction rates for Class 2 were between 0.47 (KPS*m* at *AN*) and 0.82 (TKW*m* and LAI*an* at *AN*). Under WS conditions, the prediction rates for Class 1 were between 0.56 (WSCC*an* at *GF*) and 0.85 (GY*m* at *GF*), while those for Class 2 ranged from 0.43 (KPS*m* at *AN*) to 0.78 (GY*m* at *GF*). Prediction levels between 0.40 and 0.79 for both classes were found for most traits when the hydric conditions were combined, although some of these showed prediction levels greater than 0.80 in one of the two classes: Class 1 (SM2*m AN*-FI and *GF*-FI, GY*m GF*-WS, Δ^13^C*m GF*-WS, and LAI*an GF*-FI) and Class 2 (TKW*m AN*-FI and Chl*gf AN*-FI). Prediction levels greater than 0.80 for both classes under the individual hydric conditions were only achieved for LAI*an* with prediction rates of 0.85 and 0.82 for Class 1 and Class 2, respectively (Table 3; Supplementary Table 3).

## Discussion

Despite the present study being conducted in a single year, it generated an interesting database for testing approximation methodologies. This was due to the use of a large number of cultivars/advanced lines of wheat that were grown under two contrasting hydric conditions and evaluated for a large number of traits, with spectral reflectance assessed at two developmental stages (*AN* and *GF*).

Unlike other studies, this work covers a large proportion of the SRIs reported in the remote sensing literature (Lobos and Poblete-Echeverría, 2017). In general, the regression analysis between the traits and SRIs showed an important increase in predictive potential when the experimental data from both hydric conditions were combined. Nonetheless, when compared with previous reports (Lobos et al., 2014), lower coefficients of determination were found for GY*m* and Δ^13^C*m* when the individual environments were considered.

The developmental stage at which spectral reflectance was assessed influenced the relationship between the traits and the SRIs, which has been reported previously (Marti et al., 2007; Lobos et al., 2014). The best predicted traits, GY*m* and Δ^13^C*m*, had a greater *r*^2^ at *GF*, while TKW*m* and LAI*an* had a greater *r*^2^ at *AN*; no major changes were observed for the other traits. The better prediction of GY*m* at *GF* could be related to the fact that the three main yield components (SM2, KPS, and TKW) are determined in the crop during this stage. In the case of Δ^13^C*m*, both stomatal conductance and carboxylation rate influence the carbon isotope ratio (^13^C/^12^C) in kernels, and are affected by WS in Mediterranean environments at the *GF* stage (Condon et al., 2004).

Regarding to GY*m* and Δ^13^C*m* assessed at *GF*, among the 255 SRIs tested, water indices were the ones explaining the highest variability on each environment: NWI-3 on WS and WI2 on WS+FI, while on FI WI and NWI-3 highlighted. Water indices, which combines near infrared spectra wavelengths (NIR), do not directly measure water content but instead detect the changes in leaf anatomy and cell structure that are induced by the state of hydration (Lobos et al., 2014), which influences the productivity of the plant. For GY*m*, similar results have been reported by other authors (Babar et al., 2006a; Prasad et al., 2007; Gutierrez et al., 2010; Lobos et al., 2014), while for Δ^13^C*m*, this has been reported just once (Lobos et al., 2014). This highlights the effectiveness of water indices over vegetation indices. Traits such as SM2*m*, Chl*an*, Chl*gf*, WSC*an*, and WSCC*an* correlated better with vegetation and chlorophyll SRIs, which combine visible and NIR wavelengths, but also with water indices.

Although SRIs are easy to calculate, they are limited by the use of few wavelengths. Of the multivariate regression models studied (PCR, PLSR, RR, and SVR) (Table 3, Supplementary Figure 1, and Supplementary Table 2), PCR and PLSR generally performed the same, or worse than, the SRIs. Although PLSR, which is the most popular technique used in studies of this kind, has the ability to reduce the effect of the spectral signatures collinearity through a reduction in the dimensionality of the data (Hastie et al., 2005); our results show that SRIs may perform similarly, or better, when used in plant phenotyping. On the other hand, the RR and SVR models performed the same, or better than, the SRIs (e.g., GY*m* and Δ^13^C*m* estimations increased by 8 and 10%, respectively, when using SVR). This highlights that there are multivariate regression analysis models other than PLSR that should be used in plant phenotyping to improve prediction statistics in plant breeding.

RR is a method of multivariate linear regression that includes a contraction of the multivariate model regression coefficients (Hastie et al., 2005). Although there are fewer reports of RR in remote sensing studies, this method performed better than PLSR for the estimation of plant biomass from satellite images (Cai et al., 2009) and was successfully used by Hernández et al. (2015) to predict GY in a large set of wheat genotypes. RR is known to be useful when the number of observations is much lower than the number of variables (James et al., 2013). In the present study, the number of observations is ~800, while the number of variables (reflectance values space) is around 2,000. Furthermore, RR is recognized to be an effective prediction model when there is high collinearity in the data (James et al., 2013), which is typical of spectroscopy studies where a full spectrum of reflectance is used. Our results provide empirical evidence that RR performs well in the context of spectral reflectance data, and better than more popular methods such as PLSR.

Optimization of a SVR model does not depend on the dimensionality of the input space (Smola and Vapnik, 1997). Thus, it has the ability to handle complex non-linear dependencies in high-dimensional feature spaces (Smola and Vapnik, 1997; Hastie et al., 2005), such as those modeled in this study, where it performs better than PCR and PLSR. SVR has been the subject of several comparisons in spectral studies. Wang et al. (2011) achieved more accurate estimates of rice LAI when using LS-SVM (Least Squares Support Vector Machines) rather than PLSR and MLR models. Similarly, better estimations of the nitrogen, phosphorus and potassium content in plant leaves were obtained by Zhai et al. (2013) when using SVR rather than PLSR. On the other hand, Yao et al. (2015) showed similar performance for PLSR and SVR in the prediction of nitrogen concentration in wheat leaves.

In addition to GY*m* and Δ^13^C*m* traits, improvements of between 6 and 22% were shown for the estimation of the traits SM2*m*, WSC*an*, and WSCC*an*, when assessed with spectral reflectance at *AN*, and SM2*m*, and TKW*m*, when assessed at *GF*. The best improvement in prediction was achieved for TKW*m*; this was 17 and 22% when using RR and SVR, respectively. Additionally, RR, SVR, and SRIs showed similar trends in their estimations of most traits when assessed with spectral data measured at *AN* or *GF*. The best predicted traits, GY*m* and Δ^13^C*m*, as well as SM2*m*, KPS*m*, Chl*an*, Chl*gf*, and WSCC*an*, were better-predicted using measurements of reflectance at *GF*. Meanwhile, LAI*an* was better-predicted with measurements of reflectance at *AN*, although this trait was evaluated only for well-watered plants.

The SRIs and multivariate regression models all performed much better when the data from both hydric conditions were combined (WS+FI). This situation was likely produced due to the increase in the number of samples but potentially the increase in the trait-range responses associated with the two contrasting environments was more important (Table 2). The increase in the trait-range by combining contrasting environments, and the effects on modeling improvement has been reported previously in wheat (Aparicio et al., 2002; Royo et al., 2003; Lobos et al., 2014). Because of this, special attention was paid to identification of elite genotypes (Class 2) in individual environments with a categorical approximation (PCA-LDA, PLS-DA, and *k*NN). Although the quality of a model is usually shown by its accuracy and error rate, the main difference in model performance was found to be the model's ability to identify samples from either individual (WS or FI) or combined (WS+FI) environments as Class 2, with PLS-DA being shown to be the stronger methodology (Figure 2C, Table 3). There are currently a few reports regarding the use of reflectance data and categorical methods for cereal breeding purposes (Delwiche et al., 1999, 2006, 2011; Lavine et al., 2014; Porker et al., 2017); however, all of these studies were carried out using the reflectance information from kernels or ground meal.

The selection of wheat genotypes suitable for water deficit-prone environments has traditionally been based on grain yield under irrigation conditions (yield potential) and under water deficit conditions (Araus et al., 2008; Cattivelli et al., 2008; Araus and Cairns, 2014). Even though both selected sites in this study where relatively close (70 km apart), the environmental conditions were different. Clearly the environment where the plants grew influenced the phenotype of each genotype, and therefore the spectral signature of a given genotype at each site. This could explain, in part, the differences in the estimation of each character; traits such as SM2*m*, TKW*m*, Chl*gf*, WSC*an*, and WSCC*an* showed higher predictions at FI, while GY*m*, WSC*m*, WSCC*m*, and Δ^13^C*m* were better estimated at WS.

The aim of this study was to assess the feasibility of using a categorical approximation to select featured genotypes, by comparing the performance of a large set of SRIs, multivariate regression models, and categorical models in the prediction of several traits using plant reflectance data. Even though information from only 1-year was considered, the data set used for modeling was large enough to determine the potential of each approach in plant breeding programs. Unfortunately, there are no previous studies contemplating the number of genotypes, SRIs, or the regression/categorical methods covered in this article.

Although data from additional studies and a greater number of years are needed, the present results suggest that future works oriented at plant breeding should focus on identification of elite genotypes in preference to predicting specific trait-values. The assessment of agricultural and physiological traits, such as those examined in this study, could contribute to the improvement of plant breeding programs and accelerate the selection and release of wheat genotypes/cultivars with greater adaptation to adverse environmental conditions.

## Conclusions and future perspectives

Field measurement of canopy spectral reflectance is an efficient and fast way to collect plant status information for a large number of genotypes simultaneously. Analysis of reflectance data, gathered from different hydric conditions and developmental stages, by SRIs, multivariate regression, and categorical models, allows for the prediction of agricultural and physiological traits that are related to wheat yield and water deficit adaptation. The categorical model PLS-DA proved to be a useful tool for identifying elite genotypes grown under FI or WS conditions, improving upon genotype selection based on SRIs and multivariate regression methods.

Although GY and some of the other traits evaluated in this study were predicted using SRI, multivariate regression, and categorical models, there remains a need for assessing other secondary traits that have yet to be explored in plant breeding programs.

To improve trait prediction, it will be crucial to consider other tools, such as machine learning approaches (e.g., random forest or tree-based neural networks), or include other variables that are usually assessed by remote sensing (e.g., plant temperature).

## Author contributions

AdP and IM designed the experiments, selected the germplasm, and participated on field evaluations. SR-B, FE, and AE contributed to experimental measurements and data collection. GL and AE contributed to the development of a tool for spectral analysis. CA was in charge of the implementation of modeling tools. MG and SR-B contributed to data analysis and development of the models. GL and MG were in charge of the writing up but all the authors contributed to the manuscript.

### Conflict of interest statement

The authors declare that the research was conducted in the absence of any commercial or financial relationships that could be construed as a potential conflict of interest. The reviewer LMS and handling Editor declared their shared affiliation, and the handling Editor states that the process nevertheless met the standards of a fair and objective review.
