# Supplementary material for: Assessing Wheat Traits by Spectral Reflectance: Do We Really Need to Focus on Predicted Trait-Values or Directly Identify the Elite Genotypes Group?
Source: Front Plant Sci. 2017 Mar 9;8:280. doi: 10.3389/fpls.2017.00280 (PMC5343032; doi:10.3389/fpls.2017.00280)
Supplement: Supplementary file 1 [file Table1.docx]

**SUPPLEMENTARY TABLE 1.** Traits evaluated for 384 wheat genotypes grown in different hydric conditions and classes established to develop classification models.

| **Trait**^x^ |  | **FI^z^** | |  | **WS** | |  | **WS+FI** | |
| --- | --- | --- | --- | --- | --- | --- | --- | --- | --- |
|  |  | **Class 1** | **Class 2** |  | **Class 1** | **Class 2** |  | **Class 1** | **Class 2** |
| SM2*m*^y^ | Rank | < 740.0 | ≥ 740.0 |  | < 370.0 | ≥ 370.0 |  | < 640.0 | ≥ 640.0 |
|  | n | 610 | 155 |  | 621 | 168 |  | 1236 | 319 |
| KPS*m* | Rank | < 43.7 | ≥ 43.7 |  | < 37.9 | ≥ 37.9 |  | < 41.6 | ≥ 41.6 |
|  | n | 612 | 153 |  | 629 | 160 |  | 1244 | 311 |
| TKW*m* (g) | Rank | < 54.7 | ≥ 54.7 |  | < 49.6 | ≥ 49.6 |  | < 52.5 | ≥ 52.5 |
|  | n | 612 | 153 |  | 631 | 158 |  | 1244 | 311 |
| GY*m* (ton ha^-1^) | Rank | < 10.7 | ≥ 10.7 |  | < 3.9 | ≥ 3.9 |  | < 10.0 | ≥ 10.0 |
|  | n | 612 | 153 |  | 631 | 158 |  | 1244 | 311 |
| Chl*an* (SPAD index) | Rank | < 52.0 | ≥ 52.0 |  | < 44.5 | ≥ 44.5 |  | < 50.2 | ≥ 50.2 |
|  | n | 607 | 158 |  | 629 | 160 |  | 1239 | 316 |
| Chl*gf* (SPAD index) | Rank | < 51.1 | ≥ 51.1 |  | < 41.7 | ≥ 41.7 |  | < 49.2 | ≥ 49.2 |
|  | n | 609 | 156 |  | 629 | 160 |  | 1235 | 320 |
| WSC*an* (mg g^-1^ DW) | Rank | < 175.0 | ≥ 175.0 |  | < 258.1 | ≥ 258.1 |  | < 233.5 | ≥ 233.5 |
|  | n | 612 | 153 |  | 631 | 158 |  | 1243 | 312 |
| WSC*m* (mg g^-1^ DW) | Rank | < 56.1 | ≥ 56.1 |  | < 61.2 | ≥ 61.2 |  | < 58.4 | ≥ 58.4 |
|  | n | 612 | 153 |  | 631 | 158 |  | 1244 | 311 |
| WSCC*an* (mg stem^-1^) | Rank | < 236.1 | ≥ 236.1 |  | < 512.9 | ≥ 512.9 |  | < 429.3 | ≥ 429.3 |
|  | n | 612 | 153 |  | 631 | 158 |  | 1244 | 311 |
| WSCC*m* (mg stem^-1^) | Rank | < 62.2 | ≥ 62.2 |  | < 69.2 | ≥ 69.2 |  | < 65.6 | ≥ 65.6 |
|  | n | 612 | 153 |  | 631 | 158 |  | 1244 | 311 |
| ∆^13^C*m* (‰) | Rank | < 19.2 | ≥ 19.2 |  | < 15.3 | ≥ 15.3 |  | < 18.9 | ≥ 18.9 |
|  | n | 612 | 153 |  | 631 | 158 |  | 1244 | 311 |
| LAI*an* | Rank | < 6.0 | ≥ 6.0 |  |  |  |  |  |  |
|  | n | 607 | 158 |  |  |  |  |  |  |

^x^ SM2: spikes m^-2^, KPS: kernels spike^-1^; TKW: thousand kernels weight; GY: grain yield; Chl: SPAD index; water soluble carbohydrates concentration (WSC) and content (WSCC); ∆^13^C: isotopic discrimination of ^13^C; LAI: leaf area index; n: sample number.

^y^ Trait measurement at anthesis (*an*), grain filling (*gf*), or maturity (*m*).

^z^ Hydric conditions were water stress (WS), fully irrigated (FI) and the combination (WS+FI).
