# Supplementary material for: Assessing Wheat Traits by Spectral Reflectance: Do We Really Need to Focus on Predicted Trait-Values or Directly Identify the Elite Genotypes Group?
Source: Front Plant Sci. 2017 Mar 9;8:280. doi: 10.3389/fpls.2017.00280 (PMC5343032; doi:10.3389/fpls.2017.00280)
Supplement: Supplementary file 2 [file Table2.docx]

**SUPPLEMENTARY TABLE 2.** Statistical parameters of the best SRI and regression models calculated by trait, reflectance assessment and hydric condition.

| **Trait**^v^ | **Reflectance assessment^x^** | **Hydric condition^y^** | **Spectral Reflectance**  **Indices** | | | **Regression models** | | | | | | | |
| --- | --- | --- | --- | --- | --- | --- | --- | --- | --- | --- | --- | --- | --- |
|  |  |  |  |  |  | **PCR** | | **PLSR** | | **RR** | | **SVR** | |
|  |  |  | **SRI** | **Model** | **RMSE** | **RMSE** | **AI** | **RMSE** | **AI** | **RMSE** | **AI** | **RMSE** | **AI** |
| SM2*m*^w^ | AN | WS | SR (550;670) | poly2 | 64.10 | 65.38 | 0.43 | 64.92 | 0.46 | 65.42 | 0.52 | 70.44 | 0.58 |
|  |  | FI | NRI (1510;660) | poly1 | 116.37 | 117.33 | 0.61 | 116.03 | 0.63 | 108.87 | 0.72 | 121.80 | 0.71 |
|  |  | WS+FI | MTCI (800;750;670) | poly2 | 113.93 | 145.58 | 0.73 | 119.13 | 0.85 | 96.24 | 0.92 | 97.44 | 0.92 |
|  | GF | WS | CI (415;695) | poly2 | 65.51 | 67.41 | 0.24 | 64.73 | 0.43 | 61.50 | 0.60 | 65.98 | 0.60 |
|  |  | FI | NDSI (403;830) | poly2 | 119.73 | 121.41 | 0.54 | 120.87 | 0.55 | 117.47 | 0.66 | 132.24 | 0.67 |
|  |  | WS+FI | SAVI (807;736) | poly2 | 107.96 | 119.31 | 0.86 | 106.01 | 0.89 | 93.89 | 0.92 | 96.01 | 0.92 |
| KPS*m* | AN | WS | NDSI (543;548) | poly1 | 6.90 | 7.02 | 0.15 | 7.00 | 0.20 | 7.63 | 0.53 | 7.71 | 0.51 |
|  |  | FI | RBI (695;445) | exp1 | 6.18 | 6.36 | 0.26 | 6.26 | 0.35 | 6.28 | 0.59 | 6.21 | 0.61 |
|  |  | WS+FI | NDSI (543;548) | poly2 | 6.71 | 6.87 | 0.49 | 6.82 | 0.51 | 7.01 | 0.66 | 6.72 | 0.64 |
|  | GF | WS | NDSI (933;948) | log | 6.56 | 6.86 | 0.33 | 6.82 | 0.35 | 7.10 | 0.60 | 6.88 | 0.60 |
|  |  | FI | SR (690;655) | poly2 | 6.19 | 6.37 | 0.25 | 6.24 | 0.36 | 6.61 | 0.52 | 7.26 | 0.51 |
|  |  | WS+FI | SR (960;950) | poly2 | 6.52 | 6.64 | 0.58 | 6.62 | 0.59 | 6.76 | 0.68 | 6.45 | 0.66 |
| TKW*m* | AN | WS | SR (690;655) | poly2 | 6.36 | 6.57 | 0.34 | 6.50 | 0.39 | 6.81 | 0.50 | 7.16 | 0.52 |
|  |  | FI | NRI (1510;660) | poly2 | 5.58 | 5.55 | 0.67 | 5.54 | 0.67 | 5.89 | 0.73 | 5.77 | 0.73 |
|  |  | WS+FI | NRI (1510;660) | poly2 | 6.12 | 6.34 | 0.58 | 6.33 | 0.58 | 6.38 | 0.69 | 6.17 | 0.69 |
|  | GF | WS | WI (950;900) | poly2 | 6.48 | 6.73 | 0.24 | 6.59 | 0.35 | 6.58 | 0.67 | 5.98 | 0.69 |
|  |  | FI | NRI (1510;660) | poly1 | 6.09 | 6.03 | 0.51 | 6.01 | 0.52 | 6.51 | 0.66 | 6.50 | 0.66 |
|  |  | WS+FI | WI (950;900) | poly2 | 6.55 | 6.48 | 0.53 | 6.44 | 0.55 | 6.08 | 0.74 | 5.65 | 0.74 |
| GY*m* | AN | WS | PRI * CI (570;530;760;700) | poly2 | 0.85 | 0.86 | 0.35 | 0.84 | 0.46 | 0.95 | 0.63 | 0.85 | 0.65 |
|  |  | FI | SR (440;685) | poly2 | 1.10 | 1.15 | 0.23 | 1.11 | 0.41 | 1.32 | 0.64 | 1.08 | 0.68 |
|  |  | WS+FI | NDWI (970;920) | poly1 | 1.44 | 2.08 | 0.88 | 1.93 | 0.90 | 1.15 | 0.97 | 1.10 | 0.97 |
|  | GF | WS | NDWI (970;920) | poly2 | 0.63 | 0.71 | 0.69 | 0.69 | 0.72 | 0.68 | 0.83 | 0.59 | 0.86 |
|  |  | FI | WI (900;970) | poly2 | 1.09 | 1.11 | 0.40 | 1.10 | 0.45 | 1.23 | 0.63 | 1.19 | 0.63 |
|  |  | WS+FI | WI (970;900) | poly2 | 0.95 | 1.44 | 0.95 | 1.32 | 0.96 | 0.98 | 0.98 | 0.89 | 0.98 |
| Chl*an* | AN | WS | PRI (550;531) | poly2 | 3.36 | 3.51 | 0.07 | 3.34 | 0.40 | 3.69 | 0.57 | 3.72 | 0.58 |
|  |  | FI | MCARI (700;670;550) | exp1 | 2.96 | 3.12 | 0.36 | 2.95 | 0.53 | 3.00 | 0.66 | 3.04 | 0.66 |
|  |  | WS+FI | TCARI (700;600;550;850;670) | poly2 | 3.35 | 3.76 | 0.80 | 3.59 | 0.82 | 3.44 | 0.86 | 3.36 | 0.87 |
|  | GF | WS | AI (740;887;691;698) | poly2 | 3.26 | 3.49 | 0.16 | 3.28 | 0.46 | 3.19 | 0.70 | 3.16 | 0.69 |
|  |  | FI | PRI (512;531) | exp1 | 3.08 | 3.20 | 0.23 | 3.15 | 0.33 | 3.41 | 0.53 | 3.59 | 0.54 |
|  |  | WS+FI | MTCI (800;750;670) | poly2 | 3.31 | 3.63 | 0.82 | 3.50 | 0.84 | 3.29 | 0.88 | 3.03 | 0.90 |
| Chl*gf* | AN | WS | NDSI (410;550) | poly2 | 10.11 | 10.26 | 0.12 | 10.07 | 0.27 | 10.37 | 0.48 | 10.78 | 0.46 |
|  |  | FI | MCARI (700;670;550) | poly2 | 3.28 | 3.47 | 0.36 | 3.33 | 0.50 | 3.51 | 0.60 | 3.78 | 0.58 |
|  |  | WS+FI | NDWI (970;920) | poly2 | 7.91 | 8.43 | 0.69 | 8.17 | 0.72 | 8.01 | 0.78 | 7.96 | 0.74 |
|  | GF | WS | SR (690;655) | poly2 | 10.05 | 10.13 | 0.25 | 10.08 | 0.29 | 10.67 | 0.58 | 10.12 | 0.51 |
|  |  | FI | PRI (512;531) | log | 3.41 | 3.51 | 0.28 | 3.41 | 0.42 | 3.64 | 0.60 | 3.87 | 0.60 |
|  |  | WS+FI | NDWI (970;920) | poly1 | 7.77 | 7.95 | 0.75 | 7.84 | 0.76 | 8.01 | 0.80 | 7.52 | 0.77 |
| WSC*an* | AN | WS | NDSI (1060;1118) | poly2 | 50.51 | 51.59 | 0.18 | 50.74 | 0.29 | 50.33 | 0.46 | 51.75 | 0.52 |
|  |  | FI | SAVI (807;736) | poly2 | 46.20 | 46.45 | 0.41 | 45.61 | 0.47 | 44.52 | 0.58 | 48.83 | 0.56 |
|  |  | WS+FI | MTCI (800;750;670) | poly2 | 51.00 | 58.04 | 0.58 | 55.32 | 0.66 | 47.61 | 0.81 | 48.22 | 0.80 |
|  | GF | WS | RE (670;780) | poly2 | 50.73 | 51.29 | 0.20 | 51.14 | 0.24 | 50.87 | 0.45 | 52.69 | 0.45 |
|  |  | FI | SAVI 2 (800;670) | log | 46.95 | 46.77 | 0.36 | 46.73 | 0.38 | 47.28 | 0.47 | 49.82 | 0.55 |
|  |  | WS+FI | NDSI (933;948) | poly2 | 49.55 | 52.51 | 0.72 | 51.06 | 0.75 | 49.26 | 0.81 | 47.86 | 0.80 |
| WSC*m* | AN | WS | NDTI (1650;2215) | poly1 | 22.86 | 22.69 | 0.23 | 22.61 | 0.26 | 22.77 | 0.44 | 24.56 | 0.43 |
|  |  | FI | WDVI (830;660) | poly2 | 32.59 | 32.78 | 0.11 | 32.77 | 0.12 | 32.62 | 0.16 | 33.92 | 0.28 |
|  |  | WS+FI | NDTI (1650;2215) | exp1 | 28.46 | 28.65 | 0.14 | 28.59 | 0.17 | 28.80 | 0.32 | 28.98 | 0.31 |
|  | GF | WS | NDSI (940;1122) | poly2 | 22.76 | 23.24 | 0.22 | 23.16 | 0.25 | 22.98 | 0.42 | 23.75 | 0.45 |
|  |  | FI | NDSI (1060;1118) | poly1 | 32.83 | 32.87 | 0.05 | 32.49 | 0.15 | 32.75 | 0.21 | 33.94 | 0.33 |
|  |  | WS+FI | SR (960;950) | poly2 | 28.50 | 28.77 | 0.05 | 28.63 | 0.11 | 29.13 | 0.38 | 28.52 | 0.32 |
| WSCC*an* | AN | WS | NRI (1510;660) | poly1 | 137.51 | 138.53 | 0.25 | 138.47 | 0.26 | 136.86 | 0.39 | 148.67 | 0.49 |
|  |  | FI | NRI (1510;660) | poly2 | 77.33 | 76.73 | 0.57 | 75.95 | 0.59 | 72.52 | 0.69 | 78.77 | 0.66 |
|  |  | WS+FI | MTCI (800;750;670) | poly2 | 121.77 | 140.56 | 0.64 | 125.49 | 0.77 | 111.42 | 0.84 | 114.69 | 0.83 |
|  | GF | WS | NDSI (442;438) | poly2 | 138.40 | 138.87 | 0.24 | 138.73 | 0.25 | 135.01 | 0.47 | 144.07 | 0.49 |
|  |  | FI | SAVI 2 (800;670) | exp1 | 79.35 | 79.36 | 0.48 | 79.37 | 0.49 | 79.00 | 0.58 | 84.12 | 0.61 |
|  |  | WS+FI | WI (970;900) | poly2 | 118.20 | 129.88 | 0.74 | 122.93 | 0.79 | 111.16 | 0.84 | 112.10 | 0.84 |
| WSCC*m* | AN | WS | NDTI (1650;2215) | poly2 | 33.43 | 33.56 | 0.22 | 33.35 | 0.27 | 33.47 | 0.41 | 35.40 | 0.45 |
|  |  | FI | WDVI (830;660) | poly2 | 44.16 | 44.06 | 0.10 | 44.04 | 0.12 | 43.96 | 0.20 | 45.16 | 0.29 |
|  |  | WS+FI | NDTI (1650;2215) | poly2 | 39.86 | 40.00 | 0.16 | 39.96 | 0.19 | 40.06 | 0.30 | 40.68 | 0.30 |
|  | GF | WS | NDWI (870;1260) | poly2 | 32.36 | 32.94 | 0.38 | 32.80 | 0.40 | 32.43 | 0.51 | 34.21 | 0.47 |
|  |  | FI | BI (460;660) | poly2 | 44.47 | 43.88 | 0.07 | 43.61 | 0.14 | 43.83 | 0.23 | 45.34 | 0.31 |
|  |  | WS+FI | NDSI (503;483) | log | 39.60 | 39.99 | 0.10 | 39.82 | 0.15 | 39.60 | 0.38 | 39.35 | 0.34 |
| Δ^13^C*m* | AN | WS | TCARI (700;670;550) | poly2 | 0.50 | 0.52 | 0.31 | 0.50 | 0.42 | 0.60 | 0.59 | 0.53 | 0.61 |
|  |  | FI | SIPI (800;440;680) | poly2 | 0.47 | 0.48 | 0.17 | 0.48 | 0.24 | 0.58 | 0.60 | 0.49 | 0.59 |
|  |  | WS+FI | NDWI (970;920) | poly1 | 0.84 | 1.21 | 0.87 | 1.13 | 0.90 | 0.60 | 0.98 | 0.57 | 0.98 |
|  | GF | WS | NDWI (970;920) | poly2 | 0.46 | 0.49 | 0.47 | 0.48 | 0.52 | 0.56 | 0.69 | 0.44 | 0.75 |
|  |  | FI | NDWI (970;850) | poly1 | 0.47 | 0.48 | 0.20 | 0.48 | 0.26 | 0.56 | 0.59 | 0.52 | 0.58 |
|  |  | WS+FI | WI (970;900) | poly2 | 0.56 | 0.86 | 0.95 | 0.77 | 0.96 | 0.52 | 0.98 | 0.49 | 0.98 |
| LAI*an* | AN | FI | Datt (850;710;680) | exp1 | 0.75 | 0.78 | 0.72 | 0.75 | 0.76 | 0.85 | 0.78 | 0.75 | 0.81 |
|  | GF | FI | SAVI 2 (800;670) | log | 0.82 | 0.84 | 0.63 | 0.80 | 0.70 | 0.85 | 0.77 | 0.82 | 0.77 |

^v^ SM2: spikes m^-2^, KPS: kernels spike^-1^; TKW: thousand kernels weight; GY: grain yield; Chl: SPAD index; water soluble carbohydrates concentration (WSC) and content (WSCC); ∆^13^C: isotopic discrimination of ^13^C; LAI: leaf area index.

^w^ Trait measurement at anthesis (*an*), grain filling (*gf*), or maturity (*m*).

^x^ Spectral reflectance measurement at anthesis (AN) and grain filling (GF).

^y^ Hydric conditions were water stress (WS), fully irrigated (FI) and the combination (WS+FI).
