# Supplementary material for: Assessing Wheat Traits by Spectral Reflectance: Do We Really Need to Focus on Predicted Trait-Values or Directly Identify the Elite Genotypes Group?
Source: Front Plant Sci. 2017 Mar 9;8:280. doi: 10.3389/fpls.2017.00280 (PMC5343032; doi:10.3389/fpls.2017.00280)
Supplement: Supplementary file 3 [file Table3.docx]

**SUPPLEMENTARY TABLE 3.** Statistical parameters of the categorical models calculated by trait, reflectance assessment and hydric condition.

| **Trait^v^** | **Reflectance assessment^x^** | **Hydric condition^y^** | **Model** | **Error rate cal. (Model)** | **Accuracy cal. (Model)** | **Error rate cv.**  **(Model)** | **Accuracy cv.**  **(Model)** | **Pred. rate cal.**  **(Class 1)** | **Pred. rate cv.**  **(Class 1)** | **Pred. rate cal.**  **(Class 2)** | **Pred. rate cv.**  **(Class 2)** |
| --- | --- | --- | --- | --- | --- | --- | --- | --- | --- | --- | --- |
| SM2*m*^w^ | AN | WS | PCA-LDA | 0.39 | 0.81 | 0.41 | 0.80 | 0.96 | 0.95 | 0.26 | 0.23 |
|  |  | WS | PLS-DA | 0.28 | 0.77 | 0.34 | 0.72 | 0.80 | 0.77 | 0.63 | 0.55 |
|  |  | WS | *k*NN | 0.47 | 0.75 | 0.46 | 0.75 | 0.91 | 0.91 | 0.15 | 0.17 |
|  |  | FI | PCA-LDA | 0.32 | 0.83 | 0.33 | 0.82 | 0.93 | 0.93 | 0.43 | 0.41 |
|  |  | FI | PLS-DA | 0.17 | 0.83 | 0.24 | 0.78 | 0.84 | 0.80 | 0.82 | 0.71 |
|  |  | FI | *k*NN | 0.43 | 0.74 | 0.42 | 0.75 | 0.86 | 0.86 | 0.28 | 0.30 |
|  |  | WS+FI | PCA-LDA | 0.24 | 0.86 | 0.25 | 0.85 | 0.93 | 0.93 | 0.59 | 0.57 |
|  |  | WS+FI | PLS-DA | 0.17 | 0.80 | 0.18 | 0.79 | 0.77 | 0.77 | 0.89 | 0.87 |
|  |  | WS+FI | *k*NN | 0.33 | 0.80 | 0.31 | 0.81 | 0.89 | 0.89 | 0.45 | 0.50 |
|  | GF | WS | PCA-LDA | 0.42 | 0.80 | 0.43 | 0.79 | 0.96 | 0.96 | 0.20 | 0.18 |
|  |  | WS | PLS-DA | 0.25 | 0.75 | 0.32 | 0.71 | 0.76 | 0.73 | 0.73 | 0.64 |
|  |  | WS | kNN | 0.45 | 0.70 | 0.45 | 0.70 | 0.81 | 0.82 | 0.29 | 0.29 |
|  |  | FI | PCA-LDA | 0.39 | 0.82 | 0.41 | 0.81 | 0.96 | 0.96 | 0.26 | 0.23 |
|  |  | FI | PLS-DA | 0.14 | 0.86 | 0.26 | 0.78 | 0.87 | 0.80 | 0.85 | 0.68 |
|  |  | FI | kNN | 0.44 | 0.79 | 0.42 | 0.80 | 0.95 | 0.94 | 0.18 | 0.21 |
|  |  | WS+FI | PCA-LDA | 0.29 | 0.82 | 0.29 | 0.83 | 0.90 | 0.91 | 0.51 | 0.51 |
|  |  | WS+FI | PLS-DA | 0.15 | 0.81 | 0.16 | 0.80 | 0.78 | 0.77 | 0.92 | 0.90 |
|  |  | WS+FI | kNN | 0.32 | 0.81 | 0.32 | 0.81 | 0.91 | 0.90 | 0.46 | 0.46 |
| KPS*m* | AN | WS | PCA-LDA | 0.47 | 0.80 | 0.49 | 0.79 | 0.98 | 0.98 | 0.07 | 0.04 |
|  |  | WS | PLS-DA | 0.33 | 0.70 | 0.45 | 0.63 | 0.73 | 0.68 | 0.61 | 0.43 |
|  |  | WS | *k*NN | 0.49 | 0.78 | 0.47 | 0.79 | 0.96 | 0.97 | 0.07 | 0.09 |
|  |  | FI | PCA-LDA | 0.47 | 0.80 | 0.48 | 0.79 | 0.98 | 0.98 | 0.08 | 0.06 |
|  |  | FI | PLS-DA | 0.32 | 0.74 | 0.41 | 0.67 | 0.77 | 0.72 | 0.59 | 0.47 |
|  |  | FI | *k*NN | 0.48 | 0.75 | 0.49 | 0.75 | 0.90 | 0.91 | 0.13 | 0.12 |
|  |  | WS+FI | PCA-LDA | 0.47 | 0.79 | 0.48 | 0.78 | 0.97 | 0.96 | 0.09 | 0.07 |
|  |  | WS+FI | PLS-DA | 0.31 | 0.66 | 0.32 | 0.66 | 0.65 | 0.64 | 0.73 | 0.71 |
|  |  | WS+FI | *k*NN | 0.45 | 0.71 | 0.46 | 0.71 | 0.82 | 0.82 | 0.27 | 0.27 |
|  | GF | WS | PCA-LDA | 0.48 | 0.79 | 0.48 | 0.79 | 0.97 | 0.97 | 0.06 | 0.07 |
|  |  | WS | PLS-DA | 0.36 | 0.66 | 0.37 | 0.65 | 0.68 | 0.67 | 0.59 | 0.59 |
|  |  | WS | *k*NN | 0.48 | 0.70 | 0.48 | 0.70 | 0.82 | 0.82 | 0.22 | 0.22 |
|  |  | FI | PCA-LDA | 0.48 | 0.80 | 0.49 | 0.80 | 0.99 | 0.99 | 0.05 | 0.03 |
|  |  | FI | PLS-DA | 0.38 | 0.60 | 0.41 | 0.59 | 0.59 | 0.59 | 0.64 | 0.59 |
|  |  | FI | *k*NN | 0.43 | 0.72 | 0.46 | 0.70 | 0.82 | 0.81 | 0.31 | 0.26 |
|  |  | WS+FI | PCA-LDA | 0.47 | 0.80 | 0.47 | 0.80 | 0.98 | 0.98 | 0.07 | 0.07 |
|  |  | WS+FI | PLS-DA | 0.28 | 0.68 | 0.31 | 0.66 | 0.65 | 0.64 | 0.79 | 0.73 |
|  |  | WS+FI | *k*NN | 0.41 | 0.78 | 0.41 | 0.78 | 0.91 | 0.91 | 0.27 | 0.28 |
| TKW*m* | AN | WS | PCA-LDA | 0.48 | 0.80 | 0.48 | 0.80 | 0.99 | 0.99 | 0.04 | 0.05 |
|  |  | WS | PLS-DA | 0.31 | 0.65 | 0.35 | 0.63 | 0.63 | 0.62 | 0.76 | 0.69 |
|  |  | *WS* | *kNN* | 0.46 | 0.77 | 0.45 | 0.78 | 0.93 | 0.94 | 0.15 | 0.16 |
|  |  | FI | PCA-LDA | 0.40 | 0.82 | 0.42 | 0.81 | 0.96 | 0.96 | 0.24 | 0.21 |
|  |  | FI | PLS-DA | 0.25 | 0.71 | 0.26 | 0.69 | 0.68 | 0.66 | 0.82 | 0.82 |
|  |  | FI | *k*NN | 0.42 | 0.72 | 0.42 | 0.72 | 0.82 | 0.81 | 0.34 | 0.35 |
|  |  | WS+FI | PCA-LDA | 0.38 | 0.83 | 0.39 | 0.82 | 0.97 | 0.97 | 0.27 | 0.25 |
|  |  | WS+FI | PLS-DA | 0.22 | 0.78 | 0.25 | 0.76 | 0.78 | 0.76 | 0.77 | 0.73 |
|  |  | WS+FI | *k*NN | 0.36 | 0.79 | 0.36 | 0.79 | 0.89 | 0.89 | 0.39 | 0.39 |
|  | GF | WS | PCA-LDA | 0.39 | 0.82 | 0.41 | 0.81 | 0.96 | 0.95 | 0.25 | 0.24 |
|  |  | WS | PLS-DA | 0.18 | 0.83 | 0.26 | 0.77 | 0.83 | 0.79 | 0.80 | 0.69 |
|  |  | WS | *k*NN | 0.45 | 0.71 | 0.44 | 0.71 | 0.82 | 0.81 | 0.28 | 0.30 |
|  |  | FI | PCA-LDA | 0.37 | 0.83 | 0.39 | 0.82 | 0.97 | 0.96 | 0.28 | 0.26 |
|  |  | FI | PLS-DA | 0.21 | 0.77 | 0.26 | 0.74 | 0.75 | 0.75 | 0.83 | 0.74 |
|  |  | FI | *k*NN | 0.44 | 0.71 | 0.44 | 0.72 | 0.81 | 0.82 | 0.31 | 0.30 |
|  |  | WS+FI | PCA-LDA | 0.39 | 0.82 | 0.40 | 0.81 | 0.96 | 0.96 | 0.26 | 0.24 |
|  |  | WS+FI | PLS-DA | 0.21 | 0.76 | 0.24 | 0.74 | 0.74 | 0.72 | 0.85 | 0.80 |
|  |  | WS+FI | *k*NN | 0.38 | 0.78 | 0.39 | 0.78 | 0.89 | 0.89 | 0.34 | 0.33 |
| GY*m* | AN | WS | PCA-LDA | 0.43 | 0.80 | 0.43 | 0.80 | 0.96 | 0.96 | 0.18 | 0.18 |
|  |  | WS | PLS-DA | 0.28 | 0.71 | 0.31 | 0.70 | 0.71 | 0.71 | 0.74 | 0.68 |
|  |  | WS | *k*NN | 0.46 | 0.75 | 0.45 | 0.76 | 0.89 | 0.89 | 0.18 | 0.22 |
|  |  | FI | PCA-LDA | 0.45 | 0.81 | 0.46 | 0.80 | 0.98 | 0.98 | 0.11 | 0.10 |
|  |  | FI | PLS-DA | 0.24 | 0.77 | 0.32 | 0.72 | 0.78 | 0.74 | 0.74 | 0.61 |
|  |  | FI | *k*NN | 0.48 | 0.69 | 0.47 | 0.70 | 0.81 | 0.82 | 0.22 | 0.24 |
|  |  | WS+FI | PCA-LDA | 0.27 | 0.84 | 0.28 | 0.83 | 0.91 | 0.91 | 0.56 | 0.54 |
|  |  | WS+FI | PLS-DA | 0.16 | 0.78 | 0.17 | 0.78 | 0.74 | 0.74 | 0.94 | 0.93 |
|  |  | WS+FI | *k*NN | 0.34 | 0.78 | 0.35 | 0.78 | 0.87 | 0.87 | 0.45 | 0.44 |
|  | GF | WS | PCA-LDA | 0.27 | 0.86 | 0.28 | 0.85 | 0.94 | 0.94 | 0.52 | 0.49 |
|  |  | WS | PLS-DA | 0.18 | 0.84 | 0.19 | 0.84 | 0.86 | 0.85 | 0.78 | 0.78 |
|  |  | WS | *k*NN | 0.36 | 0.82 | 0.37 | 0.82 | 0.95 | 0.95 | 0.32 | 0.32 |
|  |  | FI | PCA-LDA | 0.44 | 0.81 | 0.45 | 0.81 | 0.98 | 0.98 | 0.13 | 0.12 |
|  |  | FI | PLS-DA | 0.26 | 0.71 | 0.31 | 0.68 | 0.69 | 0.68 | 0.79 | 0.69 |
|  |  | FI | *k*NN | 0.46 | 0.78 | 0.45 | 0.79 | 0.94 | 0.94 | 0.14 | 0.18 |
|  |  | WS+FI | PCA-LDA | 0.27 | 0.84 | 0.28 | 0.83 | 0.91 | 0.91 | 0.55 | 0.53 |
|  |  | WS+FI | PLS-DA | 0.16 | 0.75 | 0.17 | 0.74 | 0.69 | 0.68 | 0.99 | 0.98 |
|  |  | WS+FI | *k*NN | 0.28 | 0.83 | 0.29 | 0.83 | 0.90 | 0.91 | 0.53 | 0.51 |
| Chl*an* | AN | WS | PCA-LDA | 0.48 | 0.80 | 0.48 | 0.79 | 0.99 | 0.98 | 0.06 | 0.06 |
|  |  | WS | PLS-DA | 0.25 | 0.75 | 0.03 | 0.71 | 0.75 | 0.73 | 0.74 | 0.65 |
|  |  | WS | *k*NN | 0.48 | 0.69 | 0.47 | 0.70 | 0.81 | 0.82 | 0.23 | 0.25 |
|  |  | FI | PCA-LDA | 0.49 | 0.79 | 0.49 | 0.79 | 0.99 | 0.99 | 0.03 | 0.03 |
|  |  | FI | PLS-DA | 0.23 | 0.77 | 0.32 | 0.70 | 0.78 | 0.72 | 0.75 | 0.63 |
|  |  | FI | *k*NN | 0.49 | 0.74 | 0.48 | 0.74 | 0.89 | 0.88 | 0.13 | 0.16 |
|  |  | WS+FI | PCA-LDA | 0.31 | 0.83 | 0.34 | 0.81 | 0.92 | 0.91 | 0.45 | 0.41 |
|  |  | WS+FI | PLS-DA | 0.18 | 0.73 | 0.18 | 0.73 | 0.67 | 0.67 | 0.97 | 0.96 |
|  |  | WS+FI | *k*NN | 0.34 | 0.79 | 0.34 | 0.79 | 0.88 | 0.88 | 0.45 | 0.45 |
|  | GF | WS | PCA-LDA | 0.41 | 0.81 | 0.42 | 0.81 | 0.97 | 0.96 | 0.21 | 0.20 |
|  |  | WS | PLS-DA | 0.28 | 0.75 | 0.31 | 0.73 | 0.77 | 0.76 | 0.68 | 0.63 |
|  |  | WS | *k*NN | 0.46 | 0.76 | 0.46 | 0.76 | 0.91 | 0.90 | 0.17 | 0.17 |
|  |  | FI | PCA-LDA | 0.45 | 0.80 | 0.48 | 0.78 | 0.98 | 0.97 | 0.11 | 0.06 |
|  |  | FI | PLS-DA | 0.32 | 0.61 | 0.34 | 0.60 | 0.57 | 0.56 | 0.79 | 0.75 |
|  |  | FI | *k*NN | 0.47 | 0.70 | 0.48 | 0.69 | 0.82 | 0.81 | 0.23 | 0.22 |
|  |  | WS+FI | PCA-LDA | 0.32 | 0.82 | 0.33 | 0.82 | 0.92 | 0.92 | 0.44 | 0.41 |
|  |  | WS+FI | PLS-DA | 0.18 | 0.73 | 0.18 | 0.73 | 0.67 | 0.67 | 0.97 | 0.97 |
|  |  | WS+FI | *k*NN | 0.36 | 0.79 | 0.35 | 0.79 | 0.88 | 0.89 | 0.40 | 0.41 |
| Chl*gf* | AN | WS | PCA-LDA | 0.46 | 0.81 | 0.46 | 0.81 | 0.99 | 0.99 | 0.10 | 0.10 |
|  |  | WS | PLS-DA | 0.31 | 0.64 | 0.34 | 0.63 | 0.60 | 0.61 | 0.78 | 0.71 |
|  |  | WS | *k*NN | 0.51 | 0.67 | 0.49 | 0.69 | 0.79 | 0.80 | 0.19 | 0.23 |
|  |  | FI | PCA-LDA | 0.45 | 0.80 | 0.45 | 0.79 | 0.97 | 0.96 | 0.14 | 0.14 |
|  |  | FI | PLS-DA | 0.27 | 0.68 | 0.28 | 0.67 | 0.64 | 0.63 | 0.81 | 0.80 |
|  |  | FI | *k*NN | 0.47 | 0.69 | 0.46 | 0.70 | 0.81 | 0.81 | 0.25 | 0.27 |
|  |  | WS+FI | PCA-LDA | 0.26 | 0.84 | 0.27 | 0.83 | 0.91 | 0.90 | 0.58 | 0.57 |
|  |  | WS+FI | PLS-DA | 0.17 | 0.76 | 0.17 | 0.76 | 0.71 | 0.71 | 0.95 | 0.95 |
|  |  | WS+FI | *k*NN | 0.29 | 0.82 | 0.30 | 0.81 | 0.89 | 0.90 | 0.52 | 0.50 |
|  | GF | WS | PCA-LDA | 0.42 | 0.81 | 0.43 | 0.81 | 0.97 | 0.96 | 0.20 | 0.18 |
|  |  | WS | PLS-DA | 0.24 | 0.77 | 0.29 | 0.75 | 0.78 | 0.77 | 0.74 | 0.66 |
|  |  | WS | *k*NN | 0.44 | 0.79 | 0.44 | 0.79 | 0.95 | 0.94 | 0.17 | 0.18 |
|  |  | FI | PCA-LDA | 0.42 | 0.81 | 0.44 | 0.80 | 0.96 | 0.96 | 0.20 | 0.16 |
|  |  | FI | PLS-DA | 0.29 | 0.69 | 0.31 | 0.68 | 0.68 | 0.68 | 0.73 | 0.71 |
|  |  | FI | *k*NN | 0.47 | 0.75 | 0.47 | 0.75 | 0.89 | 0.90 | 0.16 | 0.17 |
|  |  | WS+FI | PCA-LDA | 0.27 | 0.84 | 0.28 | 0.83 | 0.92 | 0.92 | 0.54 | 0.52 |
|  |  | WS+FI | PLS-DA | 0.17 | 0.75 | 0.17 | 0.75 | 0.70 | 0.70 | 0.96 | 0.96 |
|  |  | WS+FI | *k*NN | 0.31 | 0.81 | 0.31 | 0.81 | 0.89 | 0.90 | 0.49 | 0.48 |
| WSC*an* | AN | WS | PCA-LDA | 0.49 | 0.79 | 0.49 | 0.79 | 0.99 | 0.98 | 0.03 | 0.03 |
|  |  | WS | PLS-DA | 0.27 | 0.77 | 0.37 | 0.69 | 0.79 | 0.73 | 0.68 | 0.53 |
|  |  | WS | *k*NN | 0.48 | 0.69 | 0.47 | 0.70 | 0.80 | 0.81 | 0.25 | 0.26 |
|  |  | FI | PCA-LDA | 0.40 | 0.82 | 0.41 | 0.82 | 0.97 | 0.97 | 0.24 | 0.20 |
|  |  | FI | PLS-DA | 0.30 | 0.72 | 0.33 | 0.70 | 0.73 | 0.72 | 0.67 | 0.63 |
|  |  | FI | *k*NN | 0.46 | 0.76 | 0.46 | 0.76 | 0.91 | 0.92 | 0.16 | 0.16 |
|  |  | WS+FI | PCA-LDA | 0.37 | 0.82 | 0.39 | 0.81 | 0.94 | 0.94 | 0.31 | 0.29 |
|  |  | WS+FI | PLS-DA | 0.24 | 0.70 | 0.24 | 0.70 | 0.65 | 0.66 | 0.88 | 0.85 |
|  |  | WS+FI | *k*NN | 0.38 | 0.76 | 0.39 | 0.76 | 0.85 | 0.86 | 0.38 | 0.36 |
|  | GF | WS | PCA-LDA | 0.49 | 0.80 | 0.49 | 0.80 | 0.99 | 0.99 | 0.02 | 0.03 |
|  |  | WS | PLS-DA | 0.20 | 0.80 | 0.36 | 0.67 | 0.80 | 0.69 | 0.80 | 0.59 |
|  |  | WS | *k*NN | 0.46 | 0.75 | 0.45 | 0.76 | 0.89 | 0.90 | 0.19 | 0.19 |
|  |  | FI | PCA-LDA | 0.46 | 0.80 | 0.46 | 0.80 | 0.98 | 0.97 | 0.11 | 0.10 |
|  |  | FI | PLS-DA | 0.28 | 0.69 | 0.35 | 0.65 | 0.68 | 0.66 | 0.75 | 0.63 |
|  |  | FI | *k*NN | 0.43 | 0.74 | 0.43 | 0.74 | 0.85 | 0.85 | 0.29 | 0.29 |
|  |  | WS+FI | PCA-LDA | 0.41 | 0.80 | 0.42 | 0.79 | 0.94 | 0.93 | 0.24 | 0.22 |
|  |  | WS+FI | PLS-DA | 0.22 | 0.72 | 0.23 | 0.71 | 0.68 | 0.68 | 0.88 | 0.87 |
|  |  | WS+FI | *k*NN | 0.38 | 0.79 | 0.39 | 0.78 | 0.90 | 0.90 | 0.34 | 0.31 |
| WSC*m* | AN | WS | PCA-LDA | 0.45 | 0.80 | 0.47 | 0.78 | 0.97 | 0.95 | 0.14 | 0.11 |
|  |  | WS | PLS-DA | 0.34 | 0.71 | 0.36 | 0.70 | 0.74 | 0.73 | 0.59 | 0.55 |
|  |  | WS | *k*NN | 0.44 | 0.77 | 0.43 | 0.77 | 0.91 | 0.91 | 0.20 | 0.22 |
|  |  | FI | PCA-LDA | 0.49 | 0.80 | 0.49 | 0.80 | 0.99 | 0.99 | 0.02 | 0.01 |
|  |  | FI | PLS-DA | 0.35 | 0.68 | 0.41 | 0.65 | 0.69 | 0.68 | 0.61 | 0.51 |
|  |  | FI | *k*NN | 0.46 | 0.71 | 0.46 | 0.71 | 0.82 | 0.82 | 0.25 | 0.27 |
|  |  | WS+FI | PCA-LDA | 0.49 | 0.80 | 0.49 | 0.80 | 0.99 | 0.98 | 0.04 | 0.04 |
|  |  | WS+FI | PLS-DA | 0.38 | 0.65 | 0.40 | 0.65 | 0.68 | 0.68 | 0.55 | 0.52 |
|  |  | WS+FI | *k*NN | 0.47 | 0.70 | 0.46 | 0.70 | 0.82 | 0.82 | 0.25 | 0.26 |
|  | GF | WS | PCA-LDA | 0.44 | 0.80 | 0.45 | 0.80 | 0.97 | 0.96 | 0.15 | 0.15 |
|  |  | WS | PLS-DA | 0.28 | 0.76 | 0.33 | 0.72 | 0.78 | 0.75 | 0.67 | 0.59 |
|  |  | WS | *k*NN | 0.45 | 0.78 | 0.44 | 0.79 | 0.94 | 0.94 | 0.16 | 0.17 |
|  |  | FI | PCA-LDA | 0.48 | 0.80 | 0.48 | 0.80 | 0.99 | 0.98 | 0.05 | 0.06 |
|  |  | FI | PLS-DA | 0.36 | 0.70 | 0.39 | 0.68 | 0.73 | 0.72 | 0.54 | 0.50 |
|  |  | FI | *k*NN | 0.49 | 0.69 | 0.48 | 0.70 | 0.81 | 0.82 | 0.20 | 0.22 |
|  |  | WS+FI | PCA-LDA | 0.49 | 0.80 | 0.50 | 0.80 | 0.99 | 0.99 | 0.03 | 0.02 |
|  |  | WS+FI | PLS-DA | 0.32 | 0.71 | 0.38 | 0.67 | 0.73 | 0.70 | 0.63 | 0.54 |
|  |  | WS+FI | *k*NN | 0.47 | 0.75 | 0.47 | 0.74 | 0.89 | 0.89 | 0.17 | 0.17 |
| WSCC*an* | AN | WS | PCA-LDA | 0.47 | 0.81 | 0.48 | 0.80 | 0.99 | 0.99 | 0.06 | 0.05 |
|  |  | WS | PLS-DA | 0.38 | 0.64 | 0.40 | 0.62 | 0.66 | 0.64 | 0.58 | 0.56 |
|  |  | WS | *k*NN | 0.50 | 0.79 | 0.49 | 0.79 | 0.98 | 0.98 | 0.03 | 0.03 |
|  |  | FI | PCA-LDA | 0.36 | 0.84 | 0.39 | 0.82 | 0.98 | 0.97 | 0.31 | 0.24 |
|  |  | FI | PLS-DA | 0.29 | 0.67 | 0.29 | 0.68 | 0.65 | 0.65 | 0.78 | 0.78 |
|  |  | FI | *k*NN | 0.44 | 0.77 | 0.44 | 0.77 | 0.90 | 0.91 | 0.22 | 0.22 |
|  |  | WS+FI | PCA-LDA | 0.39 | 0.81 | 0.39 | 0.81 | 0.94 | 0.94 | 0.28 | 0.28 |
|  |  | WS+FI | PLS-DA | 0.20 | 0.71 | 0.21 | 0.71 | 0.66 | 0.66 | 0.94 | 0.92 |
|  |  | WS+FI | *k*NN | 0.37 | 0.75 | 0.36 | 0.77 | 0.84 | 0.85 | 0.41 | 0.42 |
|  | GF | WS | PCA-LDA | 0.49 | 0.80 | 0.49 | 0.80 | 0.99 | 0.99 | 0.03 | 0.03 |
|  |  | WS | PLS-DA | 0.38 | 0.58 | 0.39 | 0.58 | 0.56 | 0.56 | 0.68 | 0.66 |
|  |  | WS | *k*NN | 0.47 | 0.70 | 0.48 | 0.69 | 0.81 | 0.81 | 0.25 | 0.23 |
|  |  | FI | PCA-LDA | 0.41 | 0.82 | 0.43 | 0.81 | 0.96 | 0.96 | 0.22 | 0.18 |
|  |  | FI | PLS-DA | 0.24 | 0.73 | 0.30 | 0.70 | 0.71 | 0.70 | 0.80 | 0.70 |
|  |  | FI | *k*NN | 0.42 | 0.74 | 0.42 | 0.75 | 0.85 | 0.86 | 0.30 | 0.31 |
|  |  | WS+FI | PCA-LDA | 0.37 | 0.80 | 0.39 | 0.79 | 0.92 | 0.91 | 0.34 | 0.31 |
|  |  | WS+FI | PLS-DA | 0.19 | 0.76 | 0.20 | 0.74 | 0.72 | 0.71 | 0.91 | 0.90 |
|  |  | WS+FI | *k*NN | 0.39 | 0.77 | 0.40 | 0.77 | 0.88 | 0.88 | 0.34 | 0.31 |
| WSCC*m* | AN | WS | PCA-LDA | 0.45 | 0.80 | 0.47 | 0.79 | 0.97 | 0.97 | 0.13 | 0.09 |
|  |  | WS | PLS-DA | 0.33 | 0.63 | 0.36 | 0.63 | 0.61 | 0.62 | 0.72 | 0.66 |
|  |  | WS | *k*NN | 0.45 | 0.76 | 0.45 | 0.76 | 0.90 | 0.90 | 0.20 | 0.20 |
|  |  | FI | PCA-LDA | 0.49 | 0.80 | 0.49 | 0.80 | 0.99 | 0.99 | 0.02 | 0.01 |
|  |  | FI | PLS-DA | 0.27 | 0.71 | 0.40 | 0.64 | 0.70 | 0.67 | 0.76 | 0.54 |
|  |  | FI | *k*NN | 0.46 | 0.71 | 0.46 | 0.71 | 0.82 | 0.82 | 0.25 | 0.25 |
|  |  | WS+FI | PCA-LDA | 0.48 | 0.80 | 0.48 | 0.80 | 0.99 | 0.99 | 0.05 | 0.05 |
|  |  | WS+FI | PLS-DA | 0.36 | 0.64 | 0.37 | 0.64 | 0.64 | 0.64 | 0.64 | 0.61 |
|  |  | WS+FI | *k*NN | 0.45 | 0.71 | 0.45 | 0.71 | 0.82 | 0.82 | 0.28 | 0.28 |
|  | GF | WS | PCA-LDA | 0.45 | 0.80 | 0.46 | 0.79 | 0.97 | 0.96 | 0.14 | 0.13 |
|  |  | WS | PLS-DA | 0.24 | 0.77 | 0.33 | 0.72 | 0.78 | 0.74 | 0.75 | 0.61 |
|  |  | WS | *k*NN | 0.47 | 0.78 | 0.45 | 0.79 | 0.95 | 0.95 | 0.11 | 0.14 |
|  |  | FI | PCA-LDA | 0.46 | 0.81 | 0.48 | 0.80 | 0.99 | 0.99 | 0.08 | 0.05 |
|  |  | FI | PLS-DA | 0.22 | 0.79 | 0.36 | 0.70 | 0.79 | 0.73 | 0.76 | 0.56 |
|  |  | FI | *k*NN | 0.48 | 0.07 | 0.48 | 0.70 | 0.81 | 0.81 | 0.23 | 0.23 |
|  |  | WS+FI | PCA-LDA | 0.47 | 0.80 | 0.47 | 0.80 | 0.98 | 0.98 | 0.08 | 0.07 |
|  |  | WS+FI | PLS-DA | 0.27 | 0.74 | 0.36 | 0.69 | 0.75 | 0.71 | 0.71 | 0.58 |
|  |  | WS+FI | *k*NN | 0.48 | 0.74 | 0.47 | 0.74 | 0.88 | 0.89 | 0.16 | 0.17 |
| Δ^13^C*m* | AN | WS | PCA-LDA | 0.41 | 0.80 | 0.41 | 0.80 | 0.95 | 0.94 | 0.23 | 0.25 |
|  |  | WS | PLS-DA | 0.28 | 0.76 | 0.32 | 0.74 | 0.79 | 0.78 | 0.64 | 0.58 |
|  |  | WS | *k*NN | 0.44 | 0.77 | 0.45 | 0.77 | 0.92 | 0.91 | 0.20 | 0.20 |
|  |  | FI | PCA-LDA | 0.46 | 0.80 | 0.47 | 0.79 | 0.97 | 0.96 | 0.12 | 0.10 |
|  |  | FI | PLS-DA | 0.22 | 0.81 | 0.33 | 0.72 | 0.83 | 0.75 | 0.73 | 0.58 |
|  |  | FI | *k*NN | 0.44 | 0.80 | 0.46 | 0.79 | 0.96 | 0.96 | 0.16 | 0.13 |
|  |  | WS+FI | PCA-LDA | 0.33 | 0.82 | 0.35 | 0.80 | 0.91 | 0.90 | 0.43 | 0.40 |
|  |  | WS+FI | PLS-DA | 0.18 | 0.77 | 0.19 | 0.76 | 0.73 | 0.72 | 0.92 | 0.89 |
|  |  | WS+FI | *k*NN | 0.37 | 0.76 | 0.38 | 0.75 | 0.84 | 0.83 | 0.42 | 0.41 |
|  | GF | WS | PCA-LDA | 0.31 | 0.85 | 0.31 | 0.85 | 0.96 | 0.96 | 0.42 | 0.42 |
|  |  | WS | PLS-DA | 0.22 | 0.83 | 0.25 | 0.80 | 0.85 | 0.84 | 0.72 | 0.66 |
|  |  | WS | *k*NN | 0.42 | 0.79 | 0.39 | 0.81 | 0.94 | 0.94 | 0.22 | 0.27 |
|  |  | FI | PCA-LDA | 0.49 | 0.80 | 0.49 | 0.80 | 0.99 | 0.99 | 0.04 | 0.04 |
|  |  | FI | PLS-DA | 0.30 | 0.68 | 0.39 | 0.62 | 0.67 | 0.63 | 0.73 | 0.58 |
|  |  | FI | *k*NN | 0.43 | 0.78 | 0.44 | 0.77 | 0.91 | 0.91 | 0.23 | 0.22 |
|  |  | WS+FI | PCA-LDA | 0.32 | 0.81 | 0.34 | 0.80 | 0.90 | 0.90 | 0.46 | 0.43 |
|  |  | WS+FI | PLS-DA | 0.18 | 0.74 | 0.18 | 0.74 | 0.68 | 0.68 | 0.96 | 0.95 |
|  |  | WS+FI | *k*NN | 0.36 | 0.79 | 0.36 | 0.79 | 0.89 | 0.89 | 0.39 | 0.40 |
| LAI*an* | AN | FI | PCA-LDA | 0.21 | 0.88 | 0.21 | 0.88 | 0.95 | 0.95 | 0.63 | 0.63 |
|  |  | FI | PLS-DA | 0.14 | 0.86 | 0.17 | 0.84 | 0.86 | 0.85 | 0.85 | 0.82 |
|  |  | FI | *k*NN | 0.26 | 0.85 | 0.27 | 0.84 | 0.93 | 0.92 | 0.54 | 0.54 |
|  | GF | FI | PCA-LDA | 0.28 | 0.85 | 0.30 | 0.84 | 0.95 | 0.94 | 0.49 | 0.46 |
|  |  | FI | PLS-DA | 0.18 | 0.82 | 0.21 | 0.79 | 0.83 | 0.80 | 0.81 | 0.78 |
|  |  | FI | *k*NN | 0.40 | 0.78 | 0.39 | 0.78 | 0.90 | 0.89 | 0.30 | 0.34 |

^v^ SM2: spikes m^-2^, KPS: kernels spike^-1^; TKW: thousand kernels weight; GY: grain yield; Chl: SPAD index ; water soluble carbohydrates concentration (WSC) and content (WSCC); ∆^13^C: isotopic discrimination of ^13^C; LAI: leaf area index.

^w^ Trait measurement at anthesis (*an*), grain filling (*gf*), or maturity (*m*).

^x^ Spectral reflectance measurement at anthesis (AN) and grain filling (GF).

^y^ Hydric conditions were water stress (WS), fully irrigated (FI) and the combination (WS+FI).
