# Supplementary material for: Assessing Wheat Traits by Spectral Reflectance: Do We Really Need to Focus on Predicted Trait-Values or Directly Identify the Elite Genotypes Group?
Source: Front Plant Sci. 2017 Mar 9;8:280. doi: 10.3389/fpls.2017.00280 (PMC5343032; doi:10.3389/fpls.2017.00280)
Supplement: Supplementary file 4 [file DataSheet1.docx]

**Supplementary Figure 1**. Performance of regression models on the basis of the average of determination coefficient of cross-validation (*R^2^_cv_*) calculated to all traits and estimated by spectral reflectance at anthesis (*AN*) and grain filling (*GF*). Wheat genotypes growing under two hydric conditions (FI: fully irrigated and WS: water stress); combination of both environments (WS+FI) for modeling purposes. Vertical bars represent the standard error.
